# Docking, Binding Free Energy Calculations and In Vitro Characterization of Pyrazine Linked 2-Aminobenzamides as Novel Class I Histone Deacetylase (HDAC) Inhibitors

**DOI:** 10.3390/molecules27082526

**Published:** 2022-04-14

**Authors:** Emre F. Bülbül, Jelena Melesina, Hany S. Ibrahim, Mohamed Abdelsalam, Anita Vecchio, Dina Robaa, Matthes Zessin, Mike Schutkowski, Wolfgang Sippl

**Affiliations:** 1 Department of Medicinal Chemistry, Institute of Pharmacy, Martin-Luther University of Halle-Wittenberg, 06120 Halle (Saale), Germany; emre.bulbul@pharmazie.uni-halle.de (E.F.B.); jelenamelesina@gmail.com (J.M.); hany.ibrahim@pharmazie.uni-halle.de (H.S.I.); mohamed.abdelsalam@pharmazie.uni-halle.de (M.A.); anitavecchio95@gmail.com (A.V.); dina.robaa@pharmazie.uni-halle.de (D.R.); 2 Department of Pharmaceutical Chemistry, Faculty of Pharmacy, Egyptian Russian University, Cairo 11829, Egypt; 3 Department of Pharmaceutical Chemistry, Faculty of Pharmacy, Alexandria University, Alexandria 21521, Egypt; 4 Department of Enzymology, Institute of Biochemistry and Biotechnology, Martin-Luther-University of Halle-Wittenberg, 06120 Halle (Saale), Germany; matthes.zessin@googlemail.com (M.Z.); mike.schutkowski@biochemtech.uni-halle.de (M.S.)

**Keywords:** docking, binding free energy, 2-aminobenzamide, HDAC1, HDAC2, HDAC3 inhibitors

## Abstract

Class I histone deacetylases, HDAC1, HDAC2, and HDAC3, represent potential targets for cancer treatment. However, the development of isoform-selective drugs for these enzymes remains challenging due to their high sequence and structural similarity. In the current study, we applied a computational approach to predict the selectivity profile of developed inhibitors. Molecular docking followed by MD simulation and calculation of binding free energy was performed for a dataset of 2-aminobenzamides comprising 30 previously developed inhibitors. For each HDAC isoform, a significant correlation was found between the binding free energy values and in vitro inhibitory activities. The predictive accuracy and reliability of the best preforming models were assessed on an external test set of newly designed and synthesized inhibitors. The developed binding free-energy models are cost-effective methods and help to reduce the time required to prioritize compounds for further studies.

## 1. Introduction

Epigenetic mechanisms are controlled by chemical transformations on DNA or histone proteins [1,2], which are driven by post-translational modifications (PTM) such as methylation, sumoylation, ubiquitinylation, acetylation and others [3]. Histone deacetylases (HDACs) and histone acetyltransferases (HATs) are enzymes that are strongly involved in post-translational modifications of lysine residues of histone proteins. HDACs remove acetyl or rather acyl moieties from the N-terminal lysine residues of histones and non-histone proteins. Hence, they play a pivotal role in multiple biological processes. Due to their important role, HDACs have become promising targets for various diseases such as cancer, inflammation, parasitic infections, and neurodegenerative diseases [4,5].

So far, 18 human HDACs have been identified and subdivided into 4 classes: class I (HDAC1-3, HDAC8), class IIa (HDACs 4, 5, 7 and 9), class IIb (HDACs 6 and 10), class III (Sirtuins) and class IV (HDAC11). Class I, II and IV are called zinc-dependent HDACs, while class III is mostly called sirtuins, and they require NAD^+^ for their catalytic activity. Sirtuins are structurally different from the zinc-dependent HDACs [6].

To date, five HDAC inhibitors have been approved for the treatment of cutaneous and peripheral T-cell lymphoma and multiple myeloma. Several others are in clinical trials [7,8,9,10,11]. Most HDAC inhibitors have a common structural pharmacophore. Mostly, they contain a zinc binding group (ZBG), a linker group and a cap group [6]. The aminobenzamide scaffold is one of the most studied ZBGs. According to the released HDAC2 X-ray structure in complex with a 2-aminobenzamide derivative, this ZBG chelates the zinc ion in a bidentate manner, whereas hydrogen bonds with the neighboring histidine and catalytic tyrosine residues further stabilize the zinc coordination [12,13,14]. These inhibitors also often have a foot pocket targeting group that occupies an internal cavity characteristic to class I HDACs [15].

Since it is challenging and expensive to solve X-ray structures for all ligands of interest, molecular modelling tools are regularly applied to predict their binding modes. Reliable estimation of the protein–ligand interactions has become a promising structure-based drug design strategy. Different approaches have been developed to increase the accuracy of predicting the biological activity of small molecules [16,17,18,19,20,21]. Molecular docking and molecular dynamics studies are used to predict the binding mode of the ligands as well as the binding affinity of protein–ligand complexes. Although docking methods often correctly estimate the binding poses of ligands, their binding affinity prediction still remains a big challenge [20,22]. Thus, rescoring using binding free-energy calculations is a more popular method to rank the compounds according to their binding affinity [23]. Molecular mechanics (MM) energies combined with the Poisson-Boltzman (PB) or Generalized Born (GB) and surface area continuum solvation (SA) methods are frequently used to predict the binding free energy of the ligand. MMPBSA and MMGBSA methods are based on molecular dynamics simulations. Hence, they increase the accuracy as well as the computational time and cost [24].

In the current study, we tested whether binding free energy calculations can be used to find predictive models for a series of 2-aminobenzamide inhibitors recently reported by us [25]. We expected that the binding free energy calculations would be able to provide successful predictive models using a single frame or a small number of snapshots taken from the MD simulations. Such models repeatedly proved to be useful for the development of inhibitors of diverse epigenetic targets [26,27,28]. Thus, they can be useful for the design of novel HDAC inhibitors having 2-aminobenzamide scaffold. Additionally, such models can be used as a post-docking filter for screening large databases.

## 2. Results

### 2.1. Diversity Analysis of Studied Dataset

In this study, we focused on a particular series of inhibitors covering a reasonable biological activity range to develop a robust prediction model. The compounds chosen as a training set in this study are shown in Table 1 [25]. These compounds have a 2-aminobenzamide moiety as zinc binding group (ZBG). Either a pyrimidine or pyrazine scaffold was used as a linker group. The linker group is connected via a piperazine moiety to the cap group that includes various substituents.

**Figure 1:**
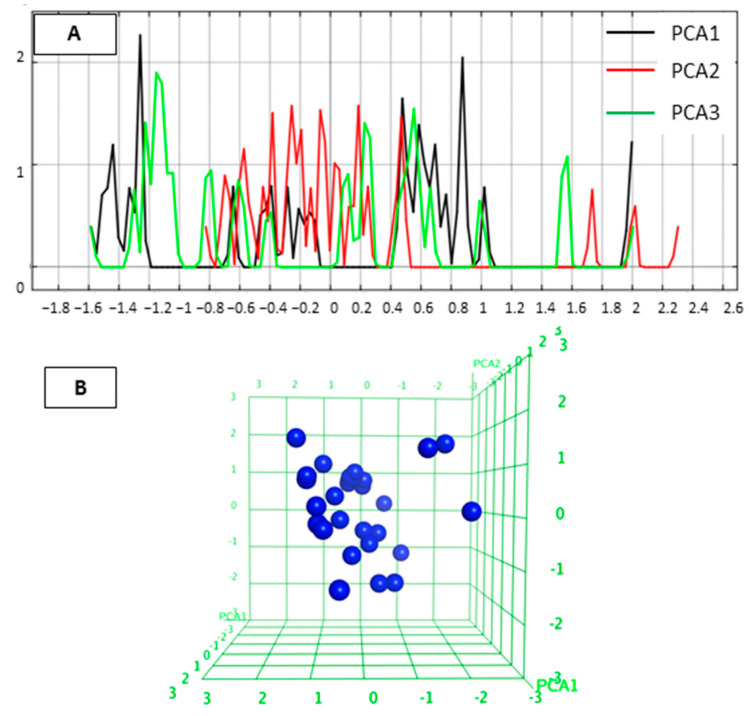
(**A**) 2D plots for visualization of the variation of three most important PCA in test set. Black color represents PCA1, red color PCA2 and green color PCA3. (**B**) 3D plot of the chemical space occupied by the training set (blue).

We first analyzed the diversity of the selected compounds. Principal component analysis (PCA) defines the chemical space of the compound data set describing the applicability domain (AD) of the established QSAR model [22,29,30]. The applicability domain can be utilized to determine the limitation of the model. In this study, PCA (PCA1, PCA2 and PCA3) calculated from the descriptors (a_acc, b_1rotN, b_ar, PEOE_VSA_POL, logP(o/w)) was used to define the AD of the training set compounds for which IC_50_ values were. The two-dimensional (2D) plots of the variations of the training set are shown in Figure 1. The PCA analysis indicated that the studied inhibitors were homogeneously distributed within the chemical space. The 3D graphical representation of the training set is shown in Figure 1B to visualize the position of the molecules.

### 2.2. Analysis of Protein-Inhibitor Complexes

**Figure 2:**
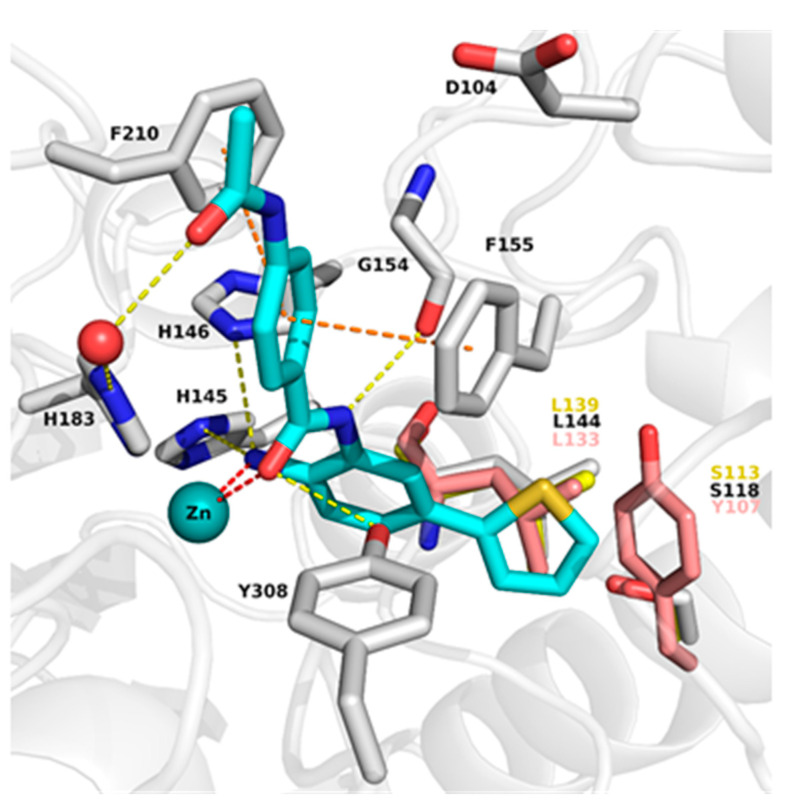
Comparison of the binding pocket of HDAC2 (white carbon atoms) with HDAC1 (yellow carbon atoms) and HDAC3 (salmon carbon atoms). Only the part of the foot pocket that differs in HDAC3 compared to HDAC1 and HDAC2 is shown for all three isoforms, and residues are labeled in the same colors as the carbon atoms. The protein backbone is shown as white ribbon. The zinc ion is shown as cyan sphere and the water molecule as red sphere. The ligand is shown as sticks with cyan carbon atoms. Protein–ligand interactions are shown as dashed lines: zinc coordination in red, hydrogen bonds in yellow, and aromatic interactions in orange.

To understand the binding mode of 2-aminobenzamide derivatives, the X-ray structures of HDAC2 in complex with similar inhibitors (PDB ID: 3MAX, 4LY1, 5IX0, 5IWG) were first analyzed. Analysis revealed that the inhibitors occupy the 11 Å long acetyl lysine substrate channel as well as the 14 Å internal cavity called the foot pocket [15]. At the bottom of the acetyl lysine substrate channel, the ligand binds in a bidentate manner to the Zn^+2^ ion through its carbonyl and free amino group. In addition, the free amino group interacts with neighboring histidine (H145 and H146) as well as tyrosine (Y308) residues. The interaction of amide-NH with glycine (G154) was observed in all HDAC2 X-ray structures in complex with 2-aminobenzamides. The aromatic linker attached to the benzamide moiety connecting the zinc binding group to the cap group is placed between two phenylalanine residues (F155 and F210) (Figure 2). The water-mediated hydrogen bond interaction with histidine (H183) was observed in HDAC2 (PDB ID 4LY1, 5IWG and 5IX0) inhibitor complexes.

The binding pocket of HDAC1 is very similar to HDAC2, whereas in HDAC3 the bulky Y107 amino acid residue situated in the foot pocket replaces the serine residues observed in HDAC1/2 (S113 and S118 in HDAC1 and HDAC2, respectively). Hence, in HDAC3 the side chain of Y107 pushes the L133 residue, causing a smaller size of the foot pocket compared to HDAC1/2. This conformational change of the L133 residue (L144 in HDAC2) may block the entry of bulky substituents into the foot pocket (Figure 2).

After careful analysis of the HDAC2 crystal structures in complex with 2-aminobenzamide, we conducted redocking (the ability to reproduce the binding mode of co-crystallized ligand) and cross-docking (the ability to correctly predict the binding mode of the other crystallized ligands) studies to find an appropriate docking protocol (Figure S1 and Table S1, Supplementary Materials). The structure PDB ID 4LY1 was chosen to dock the training set since it showed the best re- and cross-docking results, i.e., the lowest RMSD (root mean square deviation) values (Table S1). The co-crystallized ligand of HDAC2 (PDB ID 4LY1) exhibits high inhibitory activity against HDAC1 and HDAC2 [13]. Thus, the ligand from PDB ID 4LY1 was docked in HDAC1 (PDB ID: 4BKX [31]) and the complex was used for further analysis. Since this ligand does not show any inhibitory activity against HDAC3, the selective HDAC3 compound BG45 [32] was docked to HDAC3 (PDB ID: 4A69 [33]) and used for further analysis. Subsequently, the generated protein–ligand complexes were subjected to 100 ns MD simulation to check the stability of the ligands and protein–ligand interactions.

**Figure 3:**
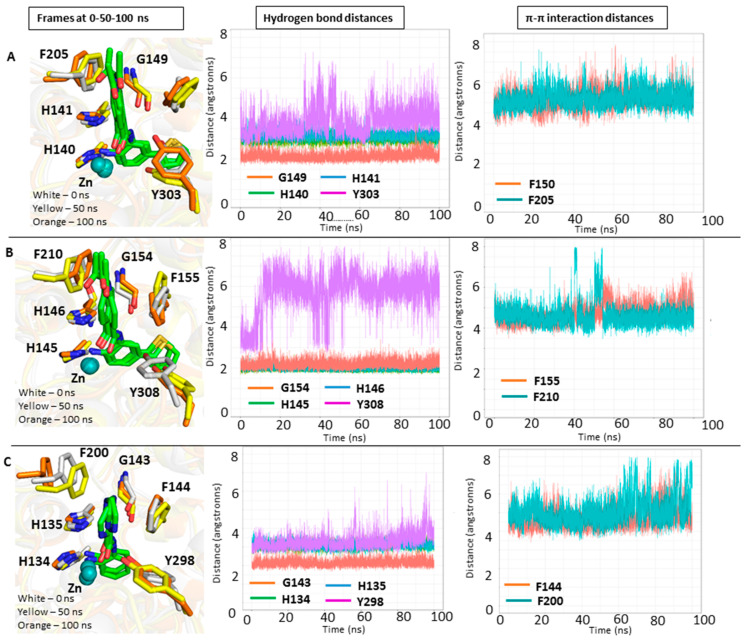
Results of the MD simulations of HDAC1, 2, 3 in complex with inhibitors. Shown are the frames of the MD simulations at 0 ns (white carbon atoms), 50 ns (yellow carbon atoms), and 100 ns (orange carbon atoms) on the left side. In the middle, distance analysis of specified hydrogen bonds between inhibitor and HDAC are shown. On the right side, the distance analysis of aromatic π–π interactions between inhibitor and HDAC are shown. HDAC1 (**A**), HDAC2 (**B**), HDAC3 (**C**). Zinc ions are shown as cyan spheres. Ligands are shown in stick representation and their carbon atoms are colored green in HDAC1, HDAC2 and HDAC3. For clarity, only relevant amino acid residues are shown.

Analysis of the MD simulation of HDAC1, HDAC2 and HDAC3-inhibitor complexes revealed that protein and ligand remained mostly stable throughout the MD simulation (Figures S2–S4, Supplementary Materials). In addition, most protein-ligand interactions were maintained throughout the 100 ns simulation time (Figure 3). The active site residues did not show significant fluctuations during the 100 ns simulations (Figure 3), with the exception of the catalytic tyrosine residue (Y303, Y308 and Y298 in HDAC1, 2 and 3, respectively) for which some flexibility was observed. More detailed analysis showed that the zinc binding group of the inhibitors maintained its bidentate chelation with the zinc ion through its free amino group and carbonyl oxygen as well as the hydrogen bond interactions with H140/145/134, H141/146/135 and G149/G154/143 in HDAC1/2/3, respectively, during the simulation. Due to the flexibility of the catalytic tyrosine residue, the observed hydrogen bond interaction with the carbonyl group was not always maintained, especially in case of HDAC1 and -2. In addition, the phenyl linker group mostly kept the aromatic interactions with the phenylalanine residues in HDAC1/2 (F150/155 and F205/210 in HDAC1/2, respectively). In HDAC3, it could be observed that the π–π interactions became less stable after 60 ns, which can be attributed to the fluctuation of F200. The thiophene ring located at the foot pocket of HDAC1 and HDAC2 stayed stable by making hydrophobic interaction with the neighboring residues in both subtypes. Meanwhile, the solvent-exposed amide cap group of the HDAC1,2 ligand underwent some small fluctuations in HDAC1 and HDAC2.

### 2.3. Docking Results of the Training Set

**Figure 4:**
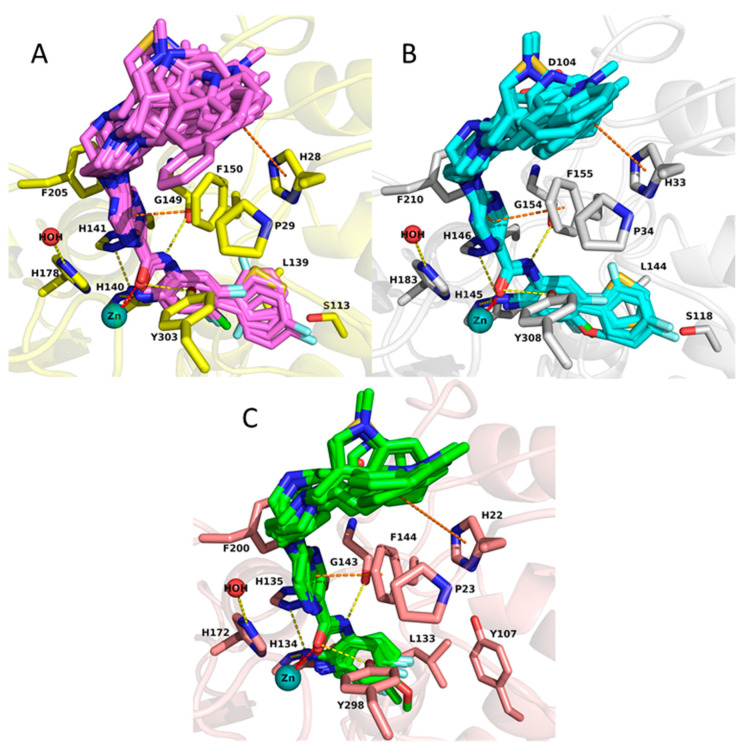
Docking poses of studied inhibitors having IC_50_ values obtained for HDAC1 (**A**), HDAC2 (**B**), HDAC3 (**C**). The carbon atoms of the shown residues are colored as following: yellow in HDAC1, white in HDAC2 and salmon in HDAC3. Zinc ions are shown as cyan spheres, water molecules as red spheres. Inhibitors are shown in stick representation and their carbon atoms are colored pink, cyan and green in HDAC1, HDAC2 and HDAC3, correspondingly. Zinc coordination is shown as red dashed lines, hydrogen bonds as yellow, and aromatic interactions as orange.

The docking results of the 30 compounds from the training set (published previously in [25]) demonstrated that they show a similar binding mode in the binding pocket of HDAC1, HDAC2 and HDAC3 (Figure 4). The compounds chelate the zinc ion and form hydrogen bonds with H140/145/134, H141/146/135, Y303/308/298, and G149/154/143 in HDAC1/2/3, respectively. Their pyrazine and pyrimidine linker groups are accommodated into the hydrophobic tunnel making π–π interactions with F150/155/144 and F205/210/200 in HDAC1/2/3, respectively. The piperazine unit shows ionic interaction with the conserved D99/104/93 in HDAC1/2/3, respectively. The cap group is surface-exposed and occupies the hydrophobic cavity, which consists of F150/155/144, P29/34/23 and H28/33/22 in HDAC1/2/3, respectively. A significant difference was observed for compounds having bulky substituents such as thiophene or phenyl groups attached to the 2-aminobenzamide moiety in the foot pocket region. These bulky substituents cannot enter into the foot pocket of HDAC3 because of the aforementioned steric hindrance.

Although the docking poses of the studied inhibitors were reasonable, a correlation between the docking scores and pIC_50_ was poor (R^2^ = 0.13 for HDAC1, R^2^ = 0.11 for HDAC2, R^2^ = 0.35 for HDAC3). The correlation between the docking scores and pIC_50_ was computed using the QSAR tool in MOE program [34]. Due to the low correlation observed between docking scores and pIC_50_, we decided to rescore the docking poses by means of binding free energy (BFE) calculations.

### 2.4. Rescoring of Docking Poses Using MM-GB/SA and MM-PB/SA

**Figure 5:**
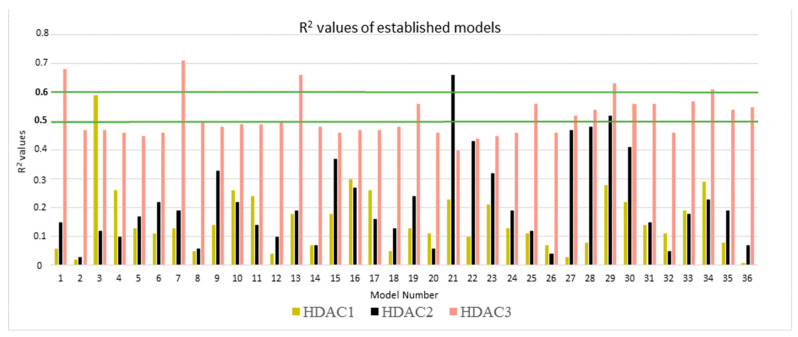
The R^2^ values of established predictive models for HDAC1 (dark yellow bars), HDAC2 (black bars) and HDAC3 (salmon bars). Green lines represent the thresholds for model selection.

The total energy was calculated using six different parameter settings and six different frame settings (see Methods part for details). In total, 108 models (36 models for each protein) were established and evaluated by computing the correlation coefficient (R^2^) between biological data and energy values (Figure 5). Only compounds that have measured IC_50_ values were considered (22 compounds for HDAC1, 23 compounds for HDAC2 and 22 compounds for HDAC3). Further details for established models are shown in Table S2.

To evaluate the obtained models, first, the energy terms showing R^2^ value less than 0.5 were skipped for HDAC1 and HDAC2. This threshold was increased to 0.6 for HDAC3 due to numerous good correlations for this isoform. In the next step, the leave-one-out cross-validation method was applied. The selected models were judged according to their R^2^, RMSE (root mean square error), Q^2^_LOO_ (leave-one-out cross-validation), and QMSE (crossed-root mean square error). The statistical parameters of the best models selected for each protein are viewed in Table 2. More details are given in Tables S3–S5.

**Figure 6:**
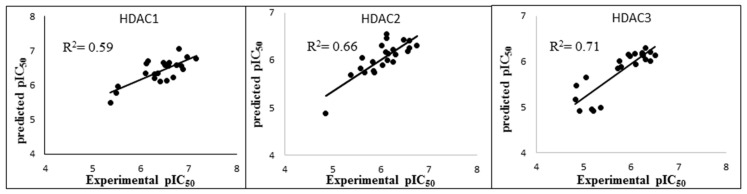
The correlation plots of the best binding free-energy models showing correlation between the predicted data and experimental data for each HDAC subtype.

In the case of HDAC1, model 3 outperformed the other models by means of the R^2^ value (Figure 5). This model was established using the total calculated energy from the GB1 model considering only frames 1–50 of the MD step. This model showed a correlation coefficient R^2^ of 0.59 and an RMSE of 0.29. The cross-validation values calculated by using the leave-one-out method were calculated as Q^2^_LOO_ 0.51 and QMSE 0.32 (Table 2). Among the established HDAC2 models, model 21 exhibited the highest R^2^ value of 0.66 (Figure 5). Model 21 was established with the combination of the GB8 model and different intervals (frames 1–50) of the MD simulation. This model showed R^2^ of 0.66, RMSE of 0.24, Q^2^_LOO_ of 0.60 and QMSE of 0.26 (Table 2). During the analysis of the HDAC3 models, five models showing R^2^ of more than 0.6 emerged (Figure 5). Based on the analysis of the R^2^ and cross-validation results, we selected Model 7 with the lowest RMSE and QMSE values and highest R^2^ and Q^2^_LOO_ values (Table 2). Thus, out of the 108 developed models, three best-performing models (one best model per HDAC subtype) were selected based on their R^2^, RMSE, Q^2^_LOO_, QMSE parameters. Their correlation plots are shown in Figure 6.

**Figure 7:**
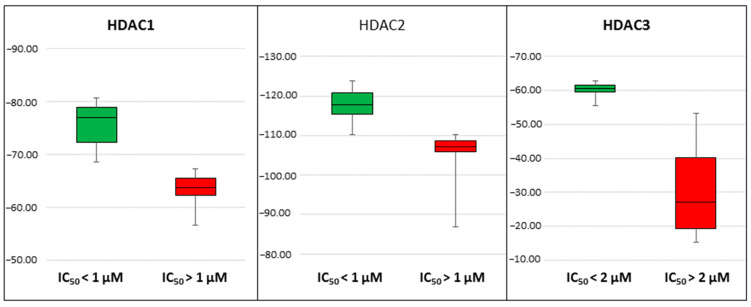
Box plots showing binding free-energy distributions (values given in kcal/mol) obtained for studied compounds.

In addition to the good correlation coefficients observed in the regression models, we noticed that the best models could discriminate the compounds in the training set (30 compounds) according to their activity. With IC_50_cut off values of 1 µM, 1 µM and 2 µM for HDAC1, 2, and 3, respectively, models could separate highly active compounds from less active ones. For HDAC1, the BFE values of all the compounds showing IC_50_ less than 1 µM were calculated lower than −68.4 kcal/mol, and only one compound with non-determined IC_50_ value (19c) crossed this threshold with a BFE value of −68.9 kcal/mol (Table S3). For HDAC2 all compounds showing IC_50_, less than 1 µM had calculated BFE values less than −110.2 kcal/mol and only two slightly less potent compounds 19m and 21a crossed this threshold (Table S4). For HDAC3, all compounds having IC_50_ less than 2 µM except 19i, 19l and 25b had calculated BFE values less than −55.4 kcal/mol, and only three compounds with non-determined IC_50_ values 19b, 19c and 19n crossed the threshold (Table S5). The discriminating power of the models is visualized in the box plots shown in Figure 7.

### 2.5. Evaluation of Novel Designed HDAC1-2-3 Inhibitors

**Figure 8:**
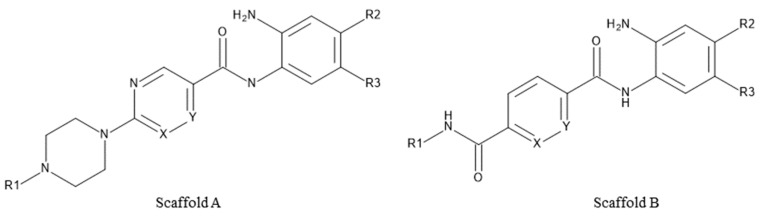
General structure of the test set molecules.

Novel derivatives of scaffold A, pyrimidine/pyrazine-piperazine scaffold, (30a, 30b, 30c and 30d) (Figure 8A, Table 3) were designed starting from the lead compound 29b in the training set in an attempt to improve selectivity for HDAC1 over HDAC2 and 3. Instead of the attachment of the long or bulky groups, a small methyl and acetyl group was added to increase the interaction with the residues at the rim of the pocket. In addition, the hydrophobicity of the secondary amine 29b was increased by the tertiary amine formation or conversion to an acetamido group.

On the other hand, in an attempt to test the impact of an inverse amide combination with hydrophobic cap group on HDAC1, 2 and 3 inhibitory activity, compounds of scaffold B (31a, 31b, 31c, Figure 8B) were designed by combining structural features of 23a, the cocrystallized ligand in HDAC2 (PDB ID: 4LY1) and entinostat. Here, a fluoro-substituted 2-aminobenzamide moiety was chosen as a foot-pocket targeting scaffold, since it previously showed a favorable effect on HDAC3 selectivity. Additionally, a substituted inverse amide scaffold was chosen as a capping group.

The established best three models for HDAC1-3 were evaluated using this test set of novel derivatives in order to assess their reliability and predictive accuracy. The test set involved seven compounds having two scaffolds (Scaffold A and Scaffold B, Figure 8, Table 3).

**Figure 9:**
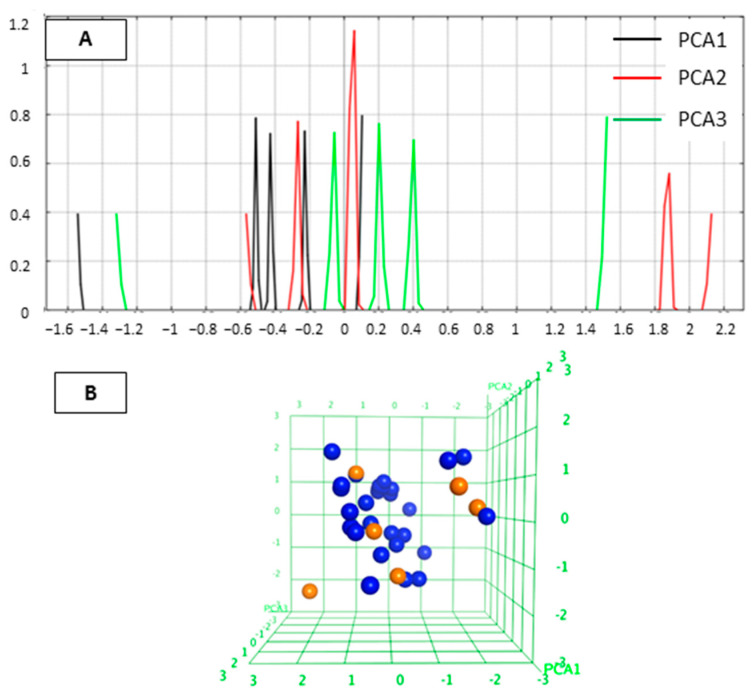
(**A**) 2D plots for visualization of the variation of three most important PCA in test set. Black color represents PCA1, red color PCA2 and green color PCA3. (**B**) 3D plot of the chemical space occupied by the training set (blue) and test set (orange).

The chemical space of the test set was analyzed in a similar way as for the training set. The 2D and 3D distribution of the test set were shown in Figure 9. The designed compounds occupy the similar PCA space with the training set and homogeneously distributed within the PCA space.

**Figure 10:**
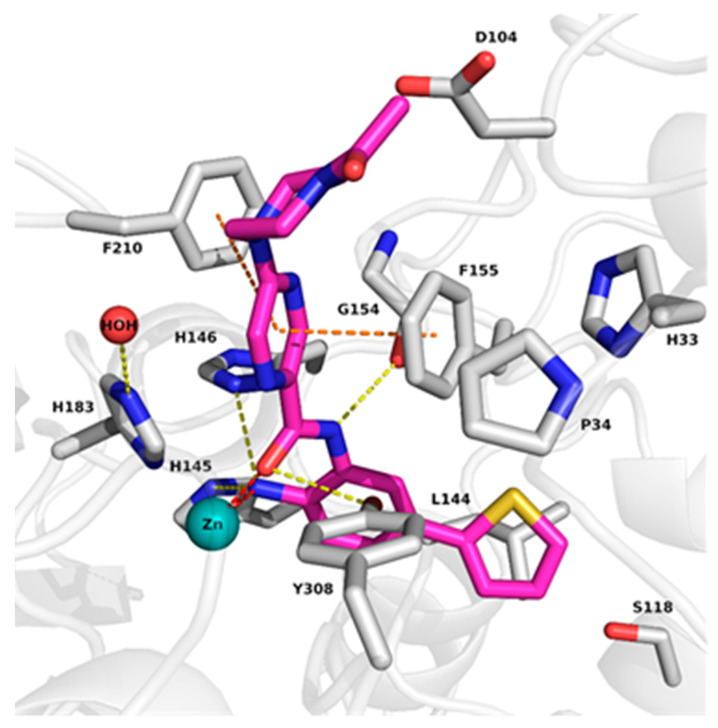
Docking pose of **30c** inHDAC2. The carbons of shown protein amino acid residues are white. Zinc ion is shown as cyan sphere, water molecule as red sphere, the ligand is shown in stick representation with magenta-colored carbon atoms. Zinc coordination is shown as red dashed lines, hydrogen bonds as yellow dashed lines and aromatic interactions as orange dashed lines.

First, the test set was docked to HDAC1-3 using the same protocol as for the training set. The expected binding modes, previously observed for reference compounds and compounds of the training set, were obtained for all compounds in HDAC1 and HDAC2, as well as for compounds 30d, 31a, 31b and 31c in HDAC3. Compounds having the 2-thienyl substituent at the foot pocket targeting group (30a, 30b, 30c) did not fit well into the pocket of HDAC3 due to the steric hindrance that is mentioned in Section 2.2. As an example, the docking pose of 30c in HDAC2 is shown in Figure 10. This compound chelated the zinc ion and established hydrogen bonds with H145, H146, Y308 and G154. The pyrimidine linker formed pi–pi interactions with F155 and F210 at the hydrophobic tunnel. Interestingly, the amide at scaffold B (31a, 31b and 31c), which is exemplified by the docking poses of 31a in Figure S5, lost the interaction with D99/104/93 in HDAC1/2/3, respectively, resulting in significantly reduced activity, although the aromatic cap group that was accommodated into hydrophobic tunnel consisted of F150/155/144, P29/34/23 and H28/33/22 in HDAC1/2/3, respectively.

The obtained docking poses were submitted to BFE calculations and the activities of the compounds were predicted using the three best models for HDAC1-3. The prediction results (Table 4, Tables S6–S8) demonstrated that the compounds in all HDAC isoforms can be correctly classified as active or weakly active/inactive taking into consideration the cutoff values established for the training set (see Section 2.4). The difference of experimental and predicted pIC_50_ was also less than <1.3 log units. Only compound 30b and 30c in HDAC1 and compound 30d in HDAC2 exhibited a bigger difference (>1 log unit) between predicted and experimentally observed pIC_50_ values, but still the compounds were correctly grouped into the activity class.

The compounds are correctly classed based on the BFE predicted activity group. Since only % inhibition was measured for the weakly active compounds from scaffold B, one can only constitute that it rightly predicts that the compounds are rather weakly active. It is not possible to deduce an IC_50_ range based solely on the % inhibition data.

## 3. Discussion

In this study, we demonstrated that the binding free energy (BFE) calculations can be used as a post-docking filter for the docking poses derived from a dataset of 2-aminobenzamide derivatives tested against HDAC1-2-3. The different setups were used to estimate the binding free energies of the protein–ligand complexes.

Redocking and cross-docking studies were performed to find the most accurate docking setup. To test the ligand flexibility and stability of the protein-inhibitor complexes we ran in addition 100 ns MD simulations. The predicted ligand-binding mode was stable and the initial ligand–protein interactions were retained throughout the whole simulation time, except for one hydrogen bond interaction with the catalytic tyrosine (Y303, Y308, Y298 in HDAC1/2/3, respectively). The increased flexibility of the catalytic tyrosine was observed in all cases. Interestingly, the conformational flexibility of the catalytic tyrosine was also reported in other HDAC2 X-ray structures in complex with a 2-substituted benzamide (PDB ID: 7KBH [35]). It might be possible that this conformational flexibility of the catalytic tyrosine can be utilized to design 6-substituted 2-aminobenzamide derivatives with pronounced selectivity in future studies.

In the second part of the study, the docking protocols for each enzyme were defined by applying the redocking and crossdocking studies. The determined docking protocols were used to understand the binding mode of the studied inhibitors. Although the docking poses correspond to the experimentally observed binding modes of similar inhibitors, the obtained docking scores unfortunately did not give a significant correlation with the experimental activities.

In the last step, the selected docking poses were rescored by performing the GPU-based binding free-energy calculations with different setups. The MM-GB/SA and MM-PB/SA values were analyzed to test whether BFE results correctly rank the compounds according to their experimental activities. Leave-one-out cross-validation studies were performed to find the best model that can explain the activity in terms of energy. We found that different energy terms gave good correlation with the biological activity in different isoforms. GB1 model and 50 frames derived from short MD for HDAC1, GB8 model and 50 frames derived from short MD for HDAC2 and GB2 model after the first minimization for HDAC3 were chosen as the best models based on the computed R^2^, RMSE, Q^2^_LOO_ and QMSE values. Additionally, the chosen models discriminated the compounds according to their activity. The rescoring of the docking poses using the GPU-based binding free energy calculations demonstrated better agreement with the experimental activity than the docking scores. The chosen models were evaluated by utilizing the test set. The selected models discriminated the inhibitors well into more active and less active, and predicted their experimentally observed activity values with high accuracy for the compounds with scaffold A. In the case of compounds with scaffold B, the model predicted them to be less active with pIC_50_ values below 6.0. Since we did not determine the exact IC_50_ value for these less-active inhibitors, a quantitative analysis of the prediction accuracy was not possible. The determined percentage of inhibition at 1 and 10 μM suggests that the compounds are active in the single-digit micromolar range and therefore less interesting. In general, the developed rescoring models can be used as primary filters to identify the most promising compounds for synthesis and in vitro testing.

## 4. Materials and Methods

### 4.1. Training Set and Diversity Analysis

The ligand data include 30 compounds recently synthesized in our group and tested against human HDAC1-3 [25]. Their chemical structures are listed in Table 1. The same numbering system was used to name the compounds as shown in the original publication by Ibrahim et al. [25]. The dataset was prepared using the Ligprep tool in Schrödinger suite with default settings [36]. Subsequently, the output of the ligand preparation step was subjected to the confgen tool [36] for the conformational enrichment by generating 64 conformers per each ligand where the output conformers were finally minimized using the OPLS3e force field [37]. Conformational enrichment is crucial to enhance the possibility to find the most likely docking poses.

The diversity of the compounds determines the limitations of the quantitative structure–activity relationship (QSAR) model. To understand the applicability domain of the studied molecules, we analyzed the most important three principal components using the principal component analysis (PCA) [38] implemented in MOE [34]. The various 2D descriptors, including number of H-bond acceptor atoms (a_acc), number of rotatable single bonds (b_1rotN), number of aromatic bonds (b_ar), total polar van der Waals surface area (PEOE_VSA_POL), logarithmic octanol/water partition coefficient (logP (o/w)), were calculated in MOE. The important descriptors that show no correlation between descriptors were selected using the Contingency module in MOE. The selected descriptors (a_acc, b_1rotN, b_ar, PEOE_VSA_POL, logP(o/w)) were applied for linear transformation by PCA. The first three principal components can explain about 100 % variation of the original space. Then, the selected first three components were used to establish a plot showing the distribution of the studied molecules.

### 4.2. Preparation of Protein-Inhibitor Complexes

X-ray crystal structures of HDAC1 (PDB ID: 4BKX [31]), HDAC2 (PDB ID: 3MAX, 4LY1, 5IX0, 5IWG [12,13,14]) and HDAC3 (PDB ID: 4A69 [33]) were downloaded from the Protein Data Bank (PDB, rcsb.org, [39]) and analyzed in MOE. The complex of HDAC2 with an inhibitor having 2-aminobenzamide scaffold (PDB ID: 4LY1, [13]) was chosen for further investigations due to the ligand’s similarity to studied compounds and good cross-docking results (Table S1). Since no crystal structure is available for the complex of HDAC1 with a 2-aminobenzamide derivative, the available crystal structure of this isoform was minimized with the ligand taken from HDAC2 to imitate induced fit effect. On the other hand, the same ligand shows no activity on HDAC3 [13]. Hence, the selective HDAC3 inhibitor BG45 was chosen and minimized with HDAC3 due to its similarity to the synthesized compounds [32]. Subsequently, protein structures were prepared using the protein preparation wizard of Schrödinger suite [36]. Hydrogen bonds and missing side chains were automatically added, and bond orders were assigned. The solvent molecules (except one conserved water molecule W617) and ions (except the catalytic Zn^+2^ ion) were removed. The protonation states at pH 7.4 and residue tautomers were optimized. Then, protein–ligand complexes were minimized using the OPLS3e force-field to remove steric clashes [37].

To check the stability of the generated complexes, 100 ns MD simulations were performed. It revealed that the protein, ligand and zinc ion were stable during the whole 100 ns simulation. Although the amide cap group of the ligand in HDAC1 and HDAC2 caused small fluctuation more than 2 Å, the other components of the ligand (ZBG, foot pocket group, linker group) were stable. In addition, the bidentate zinc coordination between zinc and ligand were conserved during 100 ns simulation (Figures S2–S4).

### 4.3. Molecular Docking

Docking studies were performed using Glide in Schrödinger Suite [36]. The active site of the proteins was defined with 10 Å radius grid box around the ligand. Standard precision (SP) mode was applied for docking. Ten docking poses were subjected for further post-docking minimization. The other settings were used as default. The docking results were visually analyzed in MOE program [34]. Before docking the novel inhibitors, we evaluated the accuracy of the docking setup by performing redocking and cross-docking of ligands from HDAC2 crystal structures (PDB IDs 3MAX, 4LY1, 5IWG and 5IX0). The docking protocol showed low RMSD values in the range between 0.2 and 1.1 Å, demonstrating that the protocol is appropriate for these proteins and ligands. In addition, molecular docking studies of the synthesized compounds were also performed for HDAC1 and HDAC3.

### 4.4. Molecular Dynamics (MD) Simulations

GPU-based MD simulations were performed using AMBER16 [40]. Each ligand was prepared in Antechamber module utilizing the semi-empirical Austin Model1 with bond charge correction (AM1-BCC) [41,42]. In addition, atom types and bond types were assigned, and residue topology files were generated. Then, protein–ligand complexes were prepared using the tLEaP module of AMBER. General amber force field (GAFF) and the Duan force field (ff03.r1) were used for ligand and protein, respectively [43,44,45]. The prepared protein–ligand complexes were solvated by TIP3P solvation model, and a margin of 10 Å. Moreover, the 12-6-4LJ ionic model was applied for the zinc ion [46]. Two minimization steps including the two substeps in each minimization were done. In the first step, 4000 iterations (2000 cycles of steepest descent and then 2000 conjugate gradient) were performed by restraining the protein residues, ligand and water molecules to their initial geometries (force constant of 10 kcal × mol^−1^ × Å^−2^) to relieve the bad contacts. In the second step, 4000 iterations (2000 cycles of steepest descent and then 2000 conjugate gradient) without restraints were applied to remove the steric clashes in the entire complex. Then, the system was heated at 300 K through 100 ps of MD while restraining the protein–ligand complex (force constant of 10 kcal × mol^−1^ × Å^−2^). The system was equilibrated with a period of 200 ps. Afterwards, a 1 ns (or 100 ns for initial complexes) MD simulation was run with a time step of 2 fs. Through the equilibration and MD step, the entire complex was kept at 300 K by the Langevin thermostat dynamics using the collision frequency of 2 ps [47]. The pressure of the system was maintained at 1 bar using isotropic pressure scaling with a relaxation time of 2 ps. A nonbonded cut-off distance was 10 Å. The particle mesh Ewald (PME) method was applied to calculate the full electrostatic energy of the system [48,49]. The SHAKE algorithm was utilized to constrain all bonds involving hydrogens [50]. Finally, after the MD job, the third energy minimization with 4000 iterations (2000 cycles of steepest descent and then 2000 conjugate gradient) was performed. The CPPTRAJ module of AMBER was used to analyze the MD snapshots.

### 4.5. Binding Free Energy (BFE) Calculations

The protein–ligand complexes were prepared as described in Section 4.2. Binding free energy (BFE) calculations were done for the docked inhibitors against HDAC1/2/3 in AMBER16 program [40]. The MMPBSA.py script was used for BFE calculations. Different radii sets (GB ^HCT^ (igb = 1), GB ^OBC^ (igb = 2), GB ^OBC2^ (igb = 5), and GBn (igb = 8) as well as PB_mbondi (mbondi) and PB_parse (parse) were tested [51,52,53,54,55]. The MM-GB(PB)/SA methods combine molecular mechanics and solvent models. In addition, six different methods listed were considered to analyze the results: (1) single frame at the first minimization step (Emin1), (2) single frame at the second minimization step (Emin2), (3) single frame at the third minimization after MD (Emin3), (4) frames 1–50 during MD (MD-1) with an interval of 5, (5) frames 51–100 during MD with an interval of 5 (MD-2), (6) frames 101–500 with an interval of 5 during MD (MD-3).

The binding free energies of protein–ligand complexes can be obtained from the difference between complex energy and the sum of the protein and ligand components (Equation (1)) [22].

ΔG_bind_ = G_complex_ − (G_protein_ + G_ligand_) (1)

The binding free energies in Equation (1) can be obtained as the sum of the gas phase energy (E_MM_), free solvation enegies (ΔG_sol_) and entropy (−TΔS) (Equation (2)) [56].

G_molecule_ = E_MM_ + ΔG_sol_ − TΔS (2)

The molecular mechanics energy (E_MM_) is calculated as the sum of E_int_ (internal), E_ele_ (electrostatic), E_vdw_ (van der Waals). The internal energies (E_int_) calculate the bond, angle and dihedral energies, while the electrostatic energies (E_ele_) take into account the interactions between atoms occurring as a result of positive and negative atomic charges. Van der Waals (E_vdw_) interactions were calculated taking into account the interactions among atoms. Solvation energies are the sum of polar solvent contributions (G_PB/GB_) and nonpolar solvent contributions (G_SA_) (Equation (3)).

ΔG_bind_ = (E_int_ + E_ele_ + E_vdw_) + (ΔG_PB/GB_ + ΔG_SA_) − TΔS (3)

The conformational entropy change (−TΔS) is often considered to determine the total binding free energies. The absolute temperature is expressed as T and entropy of the molecule as S. However, including the entropy changes to the BFE calculation increases the computational cost, and does not always improve the accuracy of the calculation [22,57]. Hence, the conformational entropy change was not included to the calculation. Only enthalpy values (ΔH) were considered to find the correlation between binding enthalpies and biological data (Equation (4)).

ΔH = (E_MM_) + (ΔG_sol_) (4)

To evaluate models, the R^2^ values of the each energy terms against biological data were computed by the Contingency tool in MOE [34].

### 4.6. Test Set

The external test data included seven newly designed and synthesized compounds that were tested against human HDAC1-3. They were used to explore the reliability and predictive power of the selected QSAR models for HDAC1-3. Their chemical structures are listed in Figure 8 and Table 3. The test dataset was prepared using the same settings as mentioned in Section 4.1.

### 4.7. Experimental Data of the Synthesized Compounds

All the experimental methods and analytical data for the newly synthesized inhibitors are provided in the Supplementary Materials (p. S24, Scheme SI and SII, [25]).

### 4.8. Biological Assay of the Synthesized Compounds

HDAC1, 2, 3 inhibitory activity for the novel inhibitors was performed as previously reported [25]. To keep the test number small, we applied a two-step screening. In a first screening, the % inhibition values for two concentrations (1 and 10 μM) were determined for all compounds. We then generated the exact IC_50_ value only for the most potent inhibitors in the training and test set with % of inhibition at 1μM above 50%.

A fluorogenic peptide derived from p53 (Ac-RHKK(Acetyl)-AMC) was used as substrate. The measurements were performed in an assay buffer (50 mM Hepes, 150 mM NaCl, 5 mM MgCl_2_, 1 mM TCEP and 0.2 mg/mL BSA, pH 7.4 adjusted with NaOH) at 37 °C. Inhibitors at different concentrations were incubated with 10 nM HDAC1, 3 nM HDAC2 or 3 nM HDAC3 (final concentration) for at least 5 min. The reaction was started with the addition of the fluorogenic substrate (20 µM final concentration) and incubated for 30 min for HDAC2 and HDAC3 and 90 min for HDAC1. The reaction was stopped with a solution of 1 mg/mL trypsin in 1 mM HCl and incubated for 1 h at 37 °C. The fluorescence intensity was recorded with an Envision 2104 Multilabel Plate Reader (PerkinElmer, Waltham, MA) with an excitation wavelength of 380 ± 8 nm and an emission wavelength of 430 ± 8 nm. The received fluorescence intensities were normalized with uninhibited reaction as 100% and the reaction without enzyme as 0%. A nonlinear regression analysis was done to determine the IC_50_ value.

## Figures and Tables

**Table 1:**
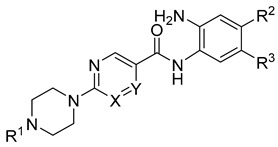
Inhibitory activity of synthesized compounds of the training set against HDAC1-3 [25].

Cpd. No.	Substituents					IC_50_ (µM) or % Inhibition at Given Concentration		
	X	Y	R1	R2	R3	HDAC1	HDAC2	HDAC3
19a	CH	N	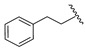	H	H	0.51 ± 0.05	0.80 ± 0.07	1.12 ± 0.07
19b	CH	N	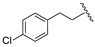	H	H	26% @ 2 µM	30% @ 2 µM	65% @ 2 µM
19c	N	CH	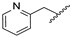	H	H	34% @ 2 µM	20% @ 2 µM	27% @ 2 µM
19d	CH	N	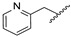	H	H	0.52 ± 0.07	1.43 ± 0.08	1.06 ± 0.04
19e	CH	N	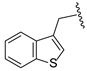	H	H	0.21 ± 0.07	0.71 ± 0.04	0.84 ± 0.03
19f	CH	N	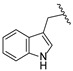	H	H	0.13 ± 0.01	0.28 ± 0.01	0.31 ± 0.01
19g	CH	N	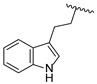	H	H	0.31 ± 0.03	0.96 ± 0.05	0.49 ± 0.06
19h	CH	N	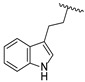	F	F	0.81 ± 0.07	0.74 ± 0.03	0.57 ± 0.02
19i	CH	N	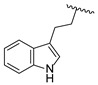	Cl	H	3.0 ± 0.2	2.7 ± 0.2	1.9 ± 0.1
19j	N	CH	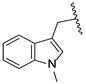	H	H	0.45 ± 0.06	0.93 ± 0.04	1.75 ± 0.06
19k	CH	N	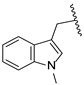	H	H	0.14 ± 0.02	0.56 ± 0.04	0.59 ± 0.03
19l	CH	N	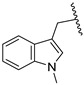	F	F	0.29 ± 0.03	0.56 ± 0.02	0.81 ± 0.05
19m	CH	N	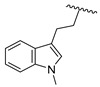	F	H	0.40 ± 0.06	1.48 ± 0.19	0.40 ± 0.02
19n	N	CH	CH_3_	H	H	5% @ 1 µM	7% @ 1 µM	13% @ 1 µM
19o	CH	N	CH_3_	H	H	27% @ 1 µM	15% @ 1 µM	30% @ 1 µM
21a	CH	N	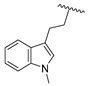	H	2-Thienyl	0.26 ± 0.01	2.47 ± 0.22	0% @ 1 µM
21b	CH	N	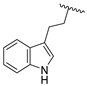	H	4-F-C_6_H_4_	0.70 ± 0.08	0.77 ± 0.06	0% @ 1 µM
21c	CH	N	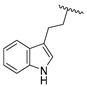	H	2-F-C_6_H_4_	0.76 ± 0.07	0.76 ± 0.04	15 ± 1
23a	CH	N	H	F	H	3.30 ± 0.18	2.17 ± 0.18	0.40 ± 0.01
23b	CH	N	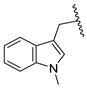	H	F	0.27 ± 0.03	0.50 ± 0.03	0.50 ± 0.02
23c	CH	N	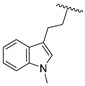	H	F	0.33 ± 0.02	1.37 ± 0.08	0.59 ± 0.04
25a	CH	N	H	Cl	H	0% @ 1 µM	0% @ 1 µM	8.7 ± 0.4
25b	CH	N	H	F	F	4.3 ± 0.3	4.2 ± 0.15	1.6 ± 0.1
27a	CH	N	H	H	CF_3_	0% @ 1 µM	0% @ 1 µM	0% @ 1 µM
27b	CH	N	H	CH_3_	H	0% @ 1 µM	0% @ 1 µM	0% @ 1 µM
27c	CH	N	H	OCH_3_	H	20.0 ± 1.0	14.0 ± 2.0	14.0 ± 1.0
29a	CH	N	CH_3_	H	3-Thienyl	0.11 ± 0.01	0.18 ± 0.06	4.4 ± 0.1
29b	CH	N	H	H	2-Thienyl	0.07 ± 0.01	0.26 ± 0.01	6.1 ± 0.7
29c	CH	N	H	H	4-F-C_6_H_4_	0.16 ± 0.03	0.34 ± 0.01	6.7 ± 0.5
29d	CH	N	CH_3_	H	2-F-C_6_H_4_	0.18 ± 0.01	0.26 ± 0.07	12.0 ± 1.0
CI994	--	--	--	--	--	37% @ 1 µM	36% @ 1 µM	32% @ 1 µM
RGFP-966	--	--	--	--	--	16 ± 2	11 ± 1	1.3 ± 0.1
MS-275	--	--	--	--	--	0.93 ± 0.1	0.95 ± 0.03	1.8 ± 0.1
Mocetinostat	--	--	--	--	--	0.33 ± 0.04	0.34 ± 0.01	0.93 ± 0.05

n.d. not determined, -- no substituents.

**Table 2:** Best performing models of HDAC1, HDAC2 and HDAC3.

Protein	N	Model Number	Solvation Model	Frame	R^2^	RMSE	Q^2^LOO	QMSE
HDAC1	22	3	GB1	MD1-50	0.59	0.29	0.51	0.32
HDAC2	23	21	GB8	MD1-50	0.66	0.24	0.60	0.26
HDAC3	22	7	GB2	Emin1	0.71	0.29	0.65	0.32

R^2^ (correlation coefficient), RMSE (root mean square error), Q^2^_LOO_ (leave-one-out cross-validation), QMSE (crossed-root mean square error), Emin1 (single frame after the first energy minimization step), MD1-50 (average energy of every fifth frame between frame 1–50 during the MD simulation).

**Table 3:** Structures of the novel inhibitors (test set).

Cpd. No.	Scaffold	Substituents				
		X	Y	R1	R2	R3
30a	A	N	CH	−CH_3_	H	2-Thienyl
30b	A	CH	N	−CH_3_	H	2-Thienyl
30c	A	CH	N	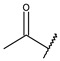	H	2-Thienyl
30d	A	CH	N	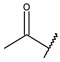	H	H
31a	B	CH	CH	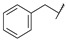	F	H
31b	B	CH	CH	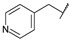	F	H
31c	B	CH	CH	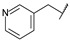	F	H

**Table 4:** Experimental and predicted activities (pIC_50_ or inhibition percent at a given concentration) of the test set compounds against HDAC1-3s.

Cpd. No.	Scaffold	Experimental HDAC1 pIC_50_	Predicted HDAC1 pIC_50_)	Difference Experimental—Predicted HDAC1 pIC_50_	Experimental HDAC2 pIC_50_	Predicted HDAC2 pIC_50_	Difference Experimental—Predicted HDAC2 pIC_50_	Experimental HDAC3 pIC_50_	Predicted HDAC3 pIC_50_
30a	A	6.49	6.55	0.06	6.21	6.00	0.21	8% @1 µM 38% @10 μM	5.06
30b	A	7.40	6.31	1.09	6.10	6.24	0.14	6% @1 µM 35% @10 μM	5.01
30c	A	7.72	6.50	1.22	5.96	5.95	0.01	4% @1 µM 30% @10 μM	4.98
30d	A	5.72	5.70	0.02	4.62	5.89	1.27	15% @1 µM 72% @10 μM	5.98
31a	B	4% @1 µM 31% @10 µM	5.58		10% @1 µM 37% @10 µM	5.52		21% @1 µM 65% @10 µM	5.81
31b	B	0% @1 µM 27% @10 µM	5.77		13% @1 µM 30% @10 µM	5.29		19% @1 µM 59% @10 µM	5.80
31c	B	6% @1 µM 36% @10 µM	5.68		14% @1 µM 51% @10 µM	5.52		25% @1 µM 66% @10 µM	5.79

## Data Availability

Not applicable.

## Supplementary Materials

The following supporting information can be downloaded at: https://www.mdpi.com/article/10.3390/molecules27082526/s1, Figure S1: Comparison of the redocking results for HDAC2 X-rays in complex with inhibitors, Figure S2: 100 ns MD results for the HDAC1-inhibitor complex, Figure S3: 100 ns MD results for the HDAC2-inhibitor complex, Figure S4: 100 ns MD results for the HDAC3-inhibitor complex, Figure S5: Docking poses of 31a in HDAC2, Table S1: RMSD results taken from the cross-docking, Table S2: R^2^ values of all models generated for HDAC1, HDAC2 and HDAC3, Table S3: The docking scores, binding free energy results of the best model and in vitro data in HDAC1, MODEL 3, Table S4: The docking scores, binding free energy results of the best model and in vitro data in HDAC2, MODEL21, Table S5: The docking scores, binding free energy results of the best model and in vitro data in HDAC3, MODEL7, Table S6: The docking scores, binding free energy results, and prediction results of the test set in HDAC1, Table S7: The docking scores, binding free energy results, and prediction results of the test set in HDAC2, Table S8: The docking scores, binding free energy results, and prediction results of the test set in HDAC3, p. S14: Synthetic methods [25]
