# Supplementary material for: Docking, Binding Free Energy Calculations and In Vitro Characterization of Pyrazine Linked 2-Aminobenzamides as Novel Class I Histone Deacetylase (HDAC) Inhibitors
Source: Molecules. 2022 Apr 14;27(8):2526. doi: 10.3390/molecules27082526 (PMC9032825; doi:10.3390/molecules27082526)
Supplement: Supplementary file 1 [file molecules-27-02526-s001.zip › molecules-1653864-supplementary.pdf]

# Docking, Binding Free Energy Calculations and in vitro Characterization of Pyrazine Linked 2-Aminobenzamides as novel Class I Histone Deacetylase (HDAC) Inhibitors

Emre F. Bülbül,[1] Jelena Melesina,[1] Hany S. Ibrahim,[1,2] Mohamed Abdelsalam,[1,3] Anita Vecchio,[1] Dina Robaa[1], Matthes Zessin,[4] Mike Schutkowski,[4] and Wolfgang Sippl[1,*]

[1]*Department of Medicinal Chemistry, Institute of Pharmacy, Martin-Luther University of Halle-Wittenberg, Halle (Saale), Germany*

[2]*Department of Pharmaceutical Chemistry, Faculty of Pharmacy, Egyptian Russian University, Badr City, Cairo 11829, Egypt*

[3]*Department of Pharmaceutical Chemistry, Faculty of Pharmacy, Alexandria University, Alexandria 21521, Egypt*

[4]*Department of Enzymology, Institute of Biochemistry and Biotechnology, Martin-Luther-University of Halle-Wittenberg, Halle (Saale), Germany*

**Table of contents**


| | Topic | Page |
|---|---|---|
| **1** | **Figure S1**. Comparison of the re-docking results for HDAC2 x-rays in complex with inhibitors. | **S3** |
| **2** | **Table S1**. RMSD results (Å) taken from the cross-docking | **S4** |
| **3** | **Figure S2**. 100ns MD results for the HDAC1-inhibitor complex | **S5** |
| **4** | **Figure S3**. 100ns MD results for the HDAC2-inhibitor complex | **S6** |
| **5** | **Figure S4**. 100ns MD results for the HDAC3-inhibitor complex | **S7** |
| **6** | **Figure S5**. Docking poses of 31a in HDAC2 | **S8** |
| **7** | **Table S2**. $R^2$ values of all models generated for HDAC1, HDAC2 and HDAC3 | **S8** |
| **8** | **Table S3.** The docking scores, binding free energy results of the best model and in vitro data in HDAC1,MODEL 3 | **S10** |
| **9** | **Table S4**. The docking scores, binding free energy results of the best model and in vitro data in HDAC2, MODEL21 | **S10** |
| **10** | **Table S5**. The docking scores, binding free energy results of the best | **S11** |

| | | |
|---|---|---|
| | model and in vitro data in HDAC3, MODEL7 | |
| **11** | **Table S6.** The docking scores, binding free energy results, and prediction results of the test set in HDAC1 | **S12** |
| **12** | **Table S7.** The docking scores, binding free energy results, and prediction results of the test set in HDAC2 | **S13** |
| **13** | **Table S8.** The docking scores, binding free energy results, and prediction results of the test set in HDAC3 | **S13** |
| **14** | **Synthetic methods** | **S14** |
| **15** | **$^{1}$H NMR and $^{13}$CNMR spectra of synthesized compounds** | **S24** |
| **16** | **References** | **S31** |

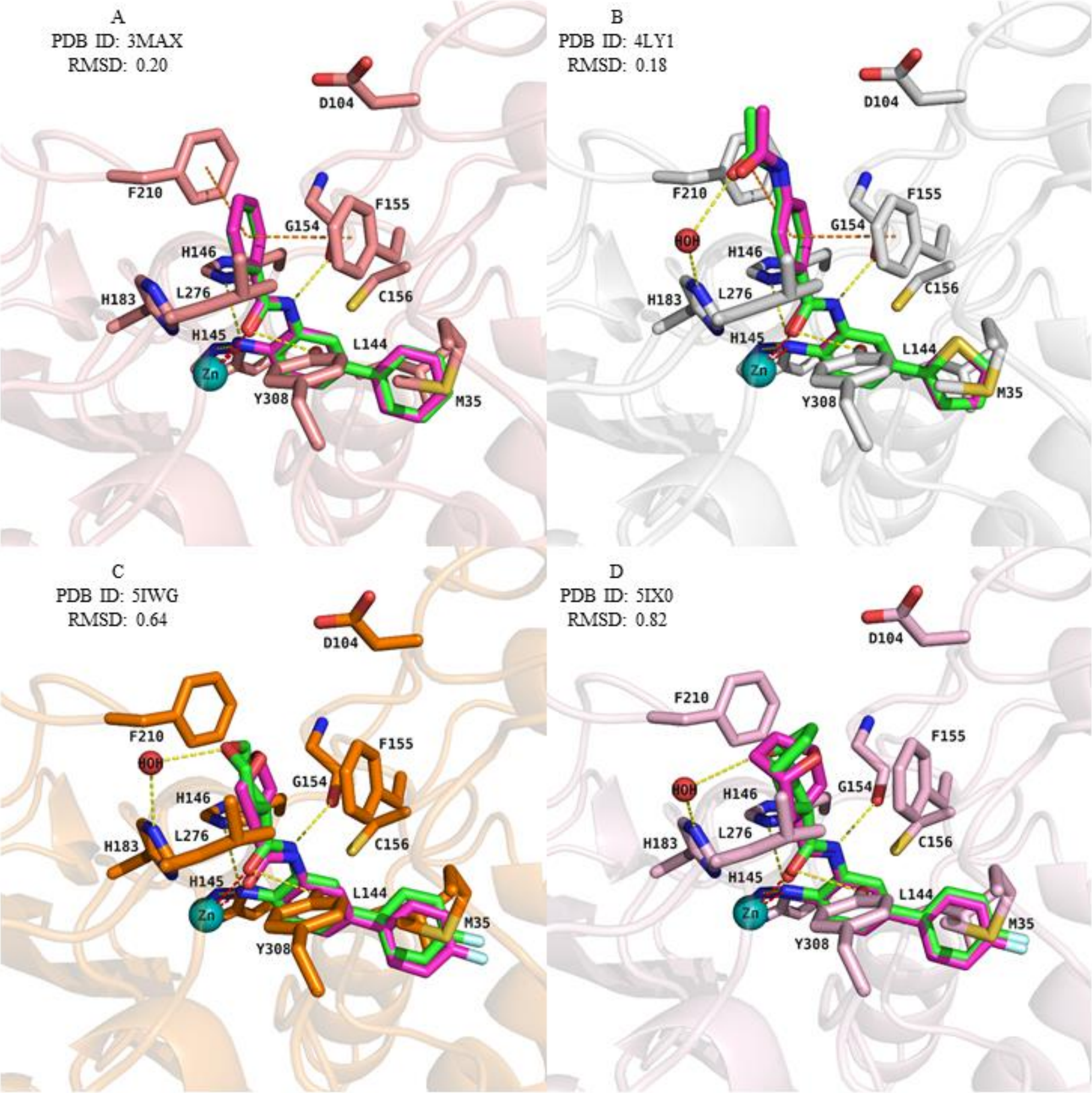


**Figure S1**. Comparison of the re-docking results for HDAC2 X-ray in complex with ligands. A) PDB ID: 3MAX, protein backbone and residues are shown as salmon, B) PDB ID: 4LY1, protein backbone and residues are shown as white color, C) PDB ID: 5IWG protein backbone and residues are shown as orange, D) PDB ID: 5IX0, protein backbone and residues are shown as pink. The re-docked poses were shown as sticks with magenta carbon atoms, and co-crystallized ligands as sticks with green carbon atoms. Zinc ion is shown as cyan sphere, water molecule as red sphere. Protein-ligand interactions are shown as dashed lines: zinc coordination in red, hydrogen bonds in yellow, and aromatic interactions in orange.

**Table S1**. Re- and crossdocking results in HDAC2 crystal structures (RMSD values in Å)

| **Inhibitors** | **PDB ID** | | | |
|---|---|---|---|---|
| | **3MAX** | **4LY1** | **5IWG** | **5IX0** |
| **3MAX** | 0.20 | 0.27 | 0.29 | 0.15 |
| **4LY1** | 1.11 | 0.18 | 0.41 | 0.21 |
| **5IWG** | 0.66 | 0.69 | 0.64 | 0.55 |
| **5IX0** | 0.96 | 0.96 | 0.97 | 0.82 |

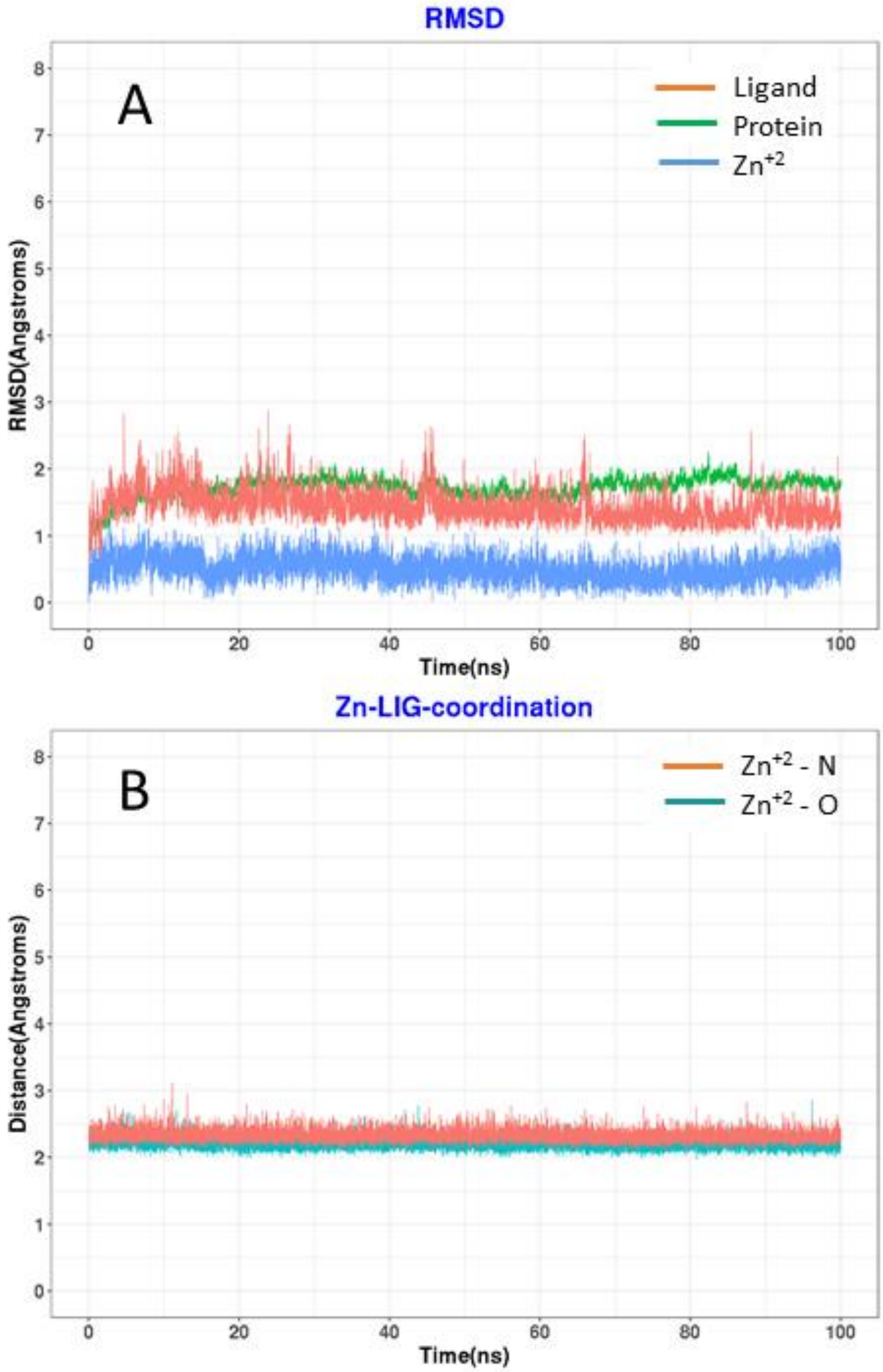


**Figure S2**. 100ns MD results for the HDAC1-inhibitor complex (PDB ID: 4BKX). A) RMSD plot for the HDAC1-ligand complex. The RMSD plot of the ligand is represented as red line, the backbone atoms of the protein as green line, and the zinc ion as blue line.

B) The distance between the zinc ion and the free amino group of the ligand (red line) and the distance between the zinc ion and carbonyl oxygen of the ligand (cyan line).

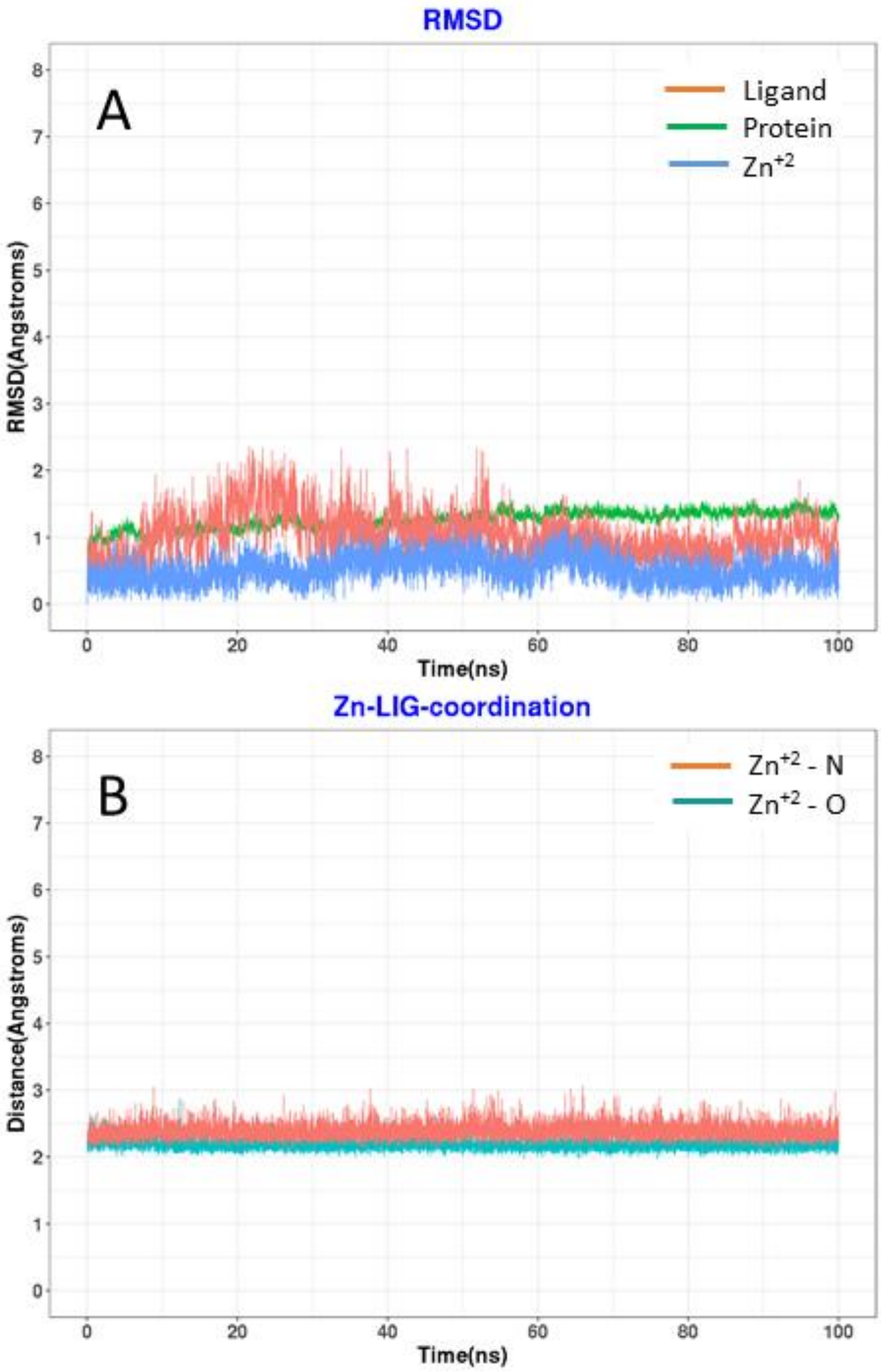


**Figure S3**. 100ns MD results for the HDAC2-inhibitor complex (PDB ID: 4LY1). A) RMSD plot for the HDAC2-ligand complex. The RMSD plot of the ligand is represented

as red line, the backbone atoms of the protein as green line, and the zinc ion as blue line. B) The distance between the zinc ion and the free amino group of the ligand (red lines) and the distance between the zinc ion and the carbonyl oxygen of ligand (cyan lines)

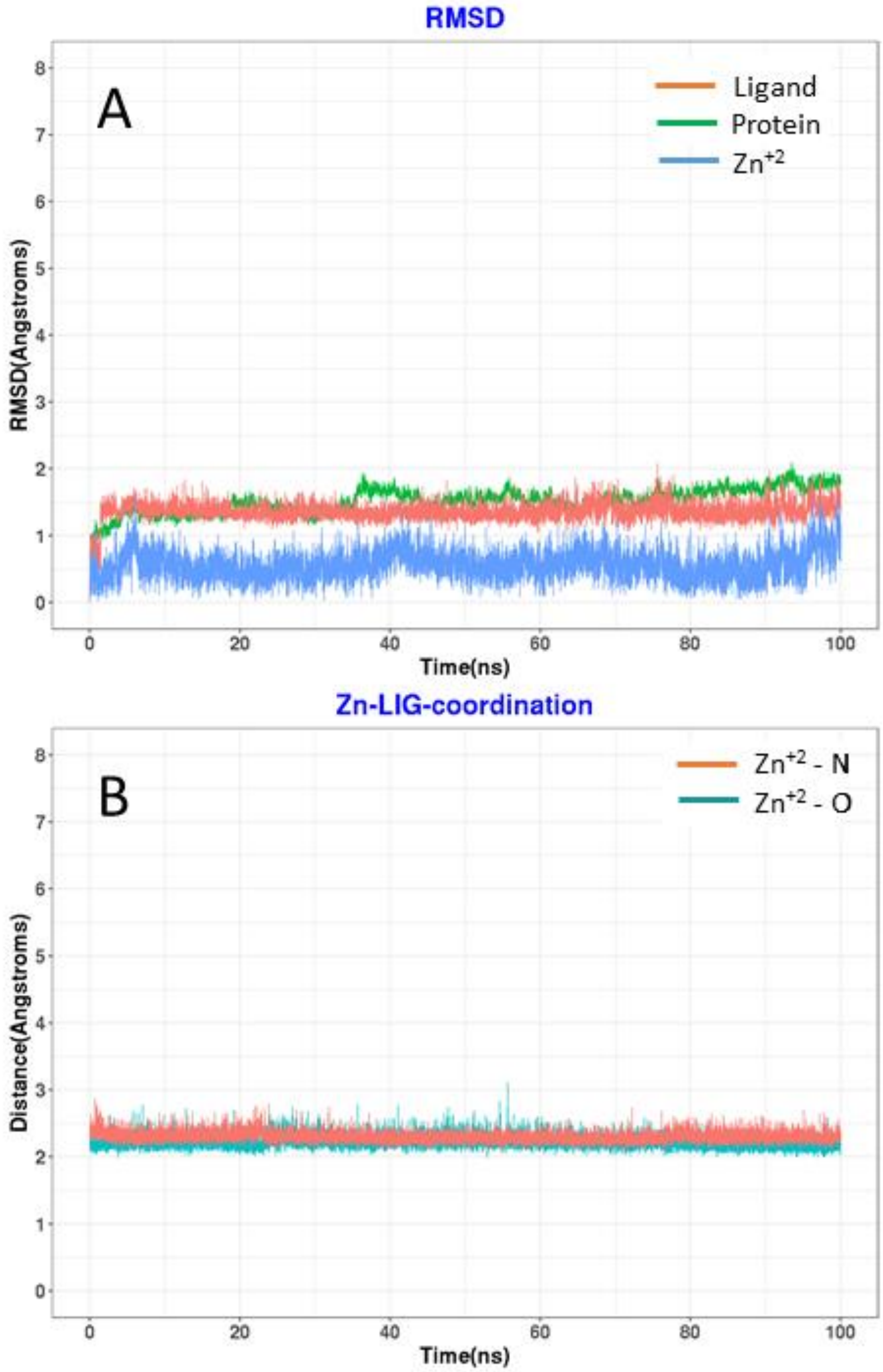


**Figure S4**. 100ns MD results for the HDAC3-inhibitor complex (PDB ID: 4A69). A) RMSD plot for the HDAC3-ligand complex. The ligand is represented as red line, the

backbone atoms of the protein as green line, and the zinc ion as blue line. B) The distance between the zinc ion and the free amino group of the ligand (red lines) and the distance between the zinc ion and the carbonyl oxygen of the ligand (cyan lines)

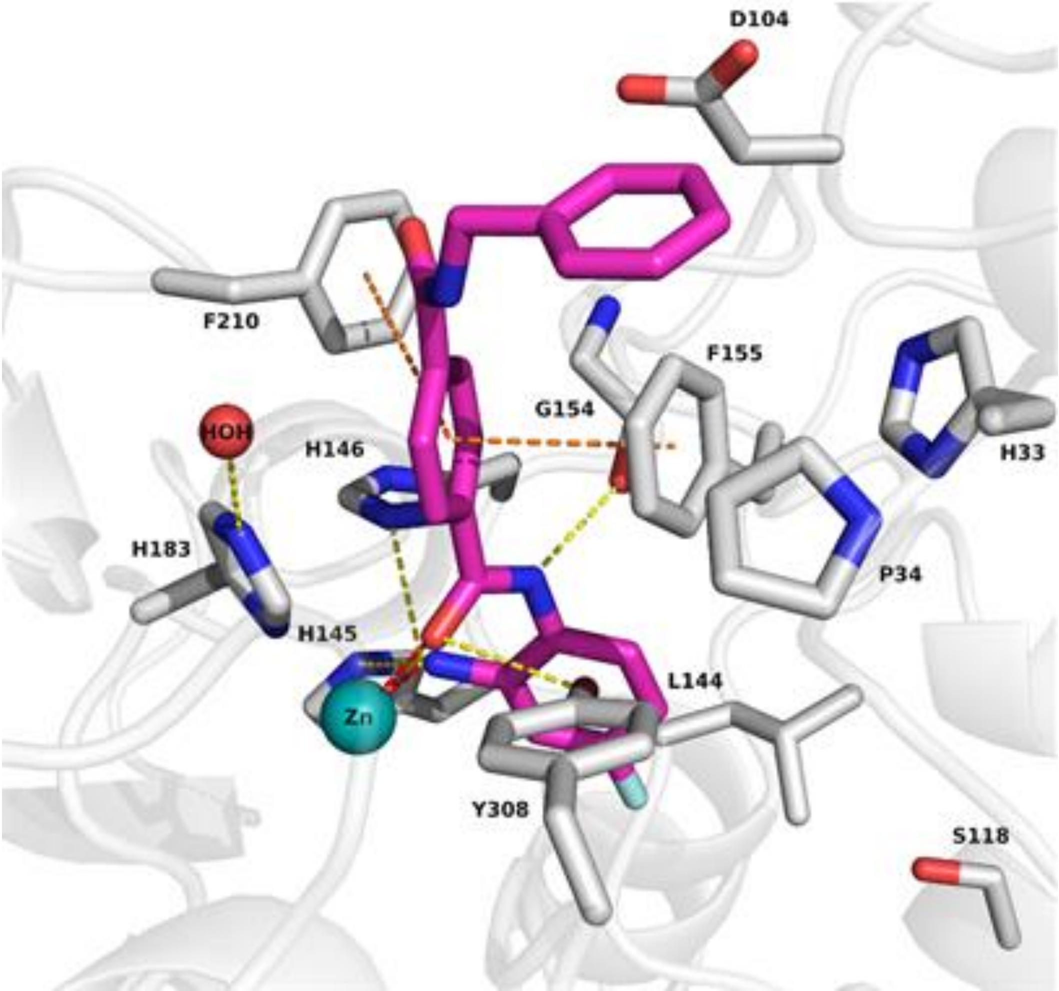


**Figure S5**. Docking pose of **31a** in HDAC2. The carbons of shown protein amino acid residues were colored as white. Zinc ion is shown as cyan spheres, water molecules as red spheres, the ligand is shown in stick representation and colored magenta. Zinc coordination is shown as red dashed lines, hydrogen bonds as yellow, and aromatic interactions as orange, electrostatic interaction as cyan.

**Table S2**. $R^2$ values of all models generated for HDAC1, HDAC2 and HDAC3

| Model | Method | Frame | $R^2$- | $R^2$- | $R^2$- |
|---|---|---|---|---|---|

| Number | | | HDAC1 n: 22 | HDAC2 n: 23 | HDAC3 n: 22 |
|---|---|---|---|---|---|
| 1 | GB1 | Emin1 | 0.06 | 0.15 | 0.68 |
| 2 | GB1 | Emin2 | 0.02 | 0.03 | 0.47 |
| 3 | GB1 | MD1-50 | 0.59 | 0.12 | 0.47 |
| 4 | GB1 | MD51-100 | 0.26 | 0.10 | 0.46 |
| 5 | GB1 | MD101-500 | 0.13 | 0.17 | 0.45 |
| 6 | GB1 | Emin3 | 0.11 | 0.22 | 0.46 |
| 7 | GB2 | Emin1 | 0.13 | 0.19 | 0.71 |
| 8 | GB2 | Emin2 | 0.05 | 0.06 | 0.50 |
| 9 | GB2 | MD1-50 | 0.14 | 0.33 | 0.48 |
| 10 | GB2 | MD51-100 | 0.26 | 0.22 | 0.49 |
| 11 | GB2 | MD101-500 | 0.24 | 0.14 | 0.49 |
| 12 | GB2 | Emin3 | 0.04 | 0.10 | 0.50 |
| 13 | GB5 | Emin1 | 0.18 | 0.19 | 0.66 |
| 14 | GB5 | Emin2 | 0.07 | 0.07 | 0.48 |
| 15 | GB5 | MD1-50 | 0.18 | 0.37 | 0.46 |
| 16 | GB5 | MD51-100 | 0.30 | 0.27 | 0.47 |
| 17 | GB5 | MD101-500 | 0.26 | 0.16 | 0.47 |
| 18 | GB5 | Emin3 | 0.05 | 0.13 | 0.48 |
| 19 | GB8 | Emin1 | 0.13 | 0.24 | 0.56 |
| 20 | GB8 | Emin2 | 0.11 | 0.06 | 0.46 |
| 21 | GB8 | MD1-50 | 0.23 | 0.66 | 0.40 |
| 22 | GB8 | MD51-100 | 0.10 | 0.43 | 0.44 |
| 23 | GB8 | MD101-500 | 0.21 | 0.32 | 0.45 |
| 24 | GB8 | Emin3 | 0.13 | 0.19 | 0.46 |
| 25 | PB-bondi | Emin1 | 0.11 | 0.12 | 0.56 |
| 26 | PB-bondi | Emin2 | 0.07 | 0.04 | 0.46 |
| 27 | PB-bondi | MD1-50 | 0.03 | 0.47 | 0.52 |
| 28 | PB-bondi | MD51-100 | 0.08 | 0.48 | 0.54 |
| 29 | PB-bondi | MD101-500 | 0.28 | 0.52 | 0.63 |
| 30 | PB-bondi | Emin3 | 0.22 | 0.41 | 0.56 |
| 31 | PB-parse | Emin1 | 0.14 | 0.15 | 0.56 |
| 32 | PB-parse | Emin2 | 0.11 | 0.05 | 0.46 |
| 33 | PB-parse | MD1-50 | 0.19 | 0.18 | 0.57 |
| 34 | PB-parse | MD51-100 | 0.29 | 0.23 | 0.61 |
| 35 | PB-parse | MD101-500 | 0.08 | 0.19 | 0.54 |
| 36 | PB-parse | Emin3 | 0.01 | 0.07 | 0.55 |

**Table S3**. The docking scores, binding free energy results of the best model and in vitro data for HDAC1, MODEL3. The compounds were colored based on the cut off values (1 µM $IC_{50}$). Green color < 1 µM, red color > 1 µM. Outlier is colored in cyan.

| HDAC1 | | | | |
|---|---|---|---|---|
| **code** | **HDAC1 $IC_{50}$ (µM) or %inhibition** | **HDAC1-$pIC_{50}$** | **Docking_score** | **GB1_MD-1-50** |
| 29b | 0.07 ± 0.01 | 7.15 | -13.05 | -81.10 |
| 29a | 0.11 ± 0.01 | 6.96 | -12.06 | -81.83 |
| 19f | 0.13 ± 0.01 | 6.89 | -9.46 | -71.75 |
| 19k | 0.14 ± 0.02 | 6.85 | -9.45 | -76.95 |
| 29c | 0.16 ± 0.03 | 6.80 | -12.33 | -86.33 |
| 29d | 0.18 ± 0.01 | 6.74 | -12.17 | -77.19 |
| 19e | 0.21 ± 0.07 | 6.68 | -9.54 | -70.59 |
| 21a | 0.26 ± 0.01 | 6.59 | -12.72 | -78.81 |
| 23b | 0.27 ± 0.03 | 6.57 | -9.83 | -76.66 |
| 19l | 0.29 ± 0.03 | 6.54 | -8.90 | -68.68 |
| 19g | 0.31 ± 0.03 | 6.51 | -10.78 | -77.26 |
| 23c | 0.33 ± 0.02 | 6.48 | -10.13 | -78.76 |
| 19m | 0.40 ± 0.06 | 6.40 | -9.11 | -68.49 |
| 19j | 0.45 ± 0.06 | 6.35 | -8.85 | -72.62 |
| 19a | 0.51 ± 0.05 | 6.29 | -10.01 | -72.55 |
| 19d | 0.52 ± 0.07 | 6.28 | -9.67 | -70.15 |
| 21b | 0.70 ± 0.08 | 6.15 | -11.68 | -79.44 |
| 21c | 0.76 ± 0.07 | 6.12 | -11.18 | -78.21 |
| 19h | 0.81 ± 0.07 | 6.09 | -10.00 | -72.69 |
| 19i | 3.0 ± 0.2 | 5.52 | -10.06 | -65.48 |
| 23a | 3.30 ± 0.18 | 5.48 | -10.09 | -62.14 |
| 25b | 4.3 ± 0.3 | 5.37 | -9.45 | -56.64 |
| 27c | 20.0 ± 1.0 | 4.7 | -11.17 | -6.25 |
| 19b | 25.8% @ 2 µM | | -10.25 | -66.93 |
| 19c | 33.9% @ 2 µM | | -9.62 | -68.88 |
| 19n | 5% @ 1 µM | | -9.80 | -64.84 |
| 19o | 27% @ 1 µM | | -9.96 | -65.31 |
| 25a | 0% @ 1 µM | | -10.67 | -62.49 |
| 27a | 0% @ 1 µM | | -10.44 | -65.72 |
| 27b | 0% @ 1 µM | | -11.33 | -56.90 |

**Table S4**. The docking scores, binding free energy results of the best model and in vitro data for HDAC2, MODEL21. The compounds were colored based on the cut off values (1 µM $IC_{50}$). Green color < 1 µM, red color > 1 µM. Outlier is colored in cyan.

| HDAC2 | | | | |
|---|---|---|---|---|
| **code** | **HDAC2 $IC_{50}$ (µM) or %inhibition** | **HDAC2-$pIC_{50}$** | **Docking_score** | **GB8_MD-1-50** |
| 29a | 0.18 ± 0.06 | 6.74 | -14.10 | -119,64 |
| 29b | 0.26 ± 0.01 | 6.59 | -14.88 | -118,62 |
| 29d | 0.26 ± 0.07 | 6.59 | -14.38 | -122,05 |
| 19f | 0.28 ± 0.01 | 6.55 | -11.30 | -116,99 |
| 29c | 0.34 ± 0.01 | 6.47 | -14.56 | -122,36 |
| 23b | 0.50 ± 0.03 | 6.30 | -11.17 | -115,33 |
| 19k | 0.56 ± 0.04 | 6.25 | -11.59 | -117,82 |
| 19l | 0.56 ± 0.02 | 6.25 | -10.94 | -111,69 |
| 19e | 0.71 ± 0.04 | 6.15 | -11.24 | -115,59 |
| 19h | 0.74 ± 0.03 | 6.13 | -11.37 | -112,49 |
| 21c | 0.76 ± 0.04 | 6.12 | -14.42 | -123,37 |
| 21b | 0.77 ± 0.06 | 6.11 | -13.72 | -125,34 |
| 19a | 0.80 ± 0.07 | 6.10 | -11.80 | -116,37 |
| 19j | 0.93 ± 0.04 | 6.03 | -11.00 | -110,26 |
| 19g | 0.96 ± 0.05 | 6.02 | -11.82 | -119,63 |
| 23c | 1.37 ± 0.08 | 5.86 | -11.71 | -106,59 |
| 19d | 1.43 ± 0.08 | 5.84 | -11.07 | -107,25 |
| 19m | 1.48 ± 0.19 | 5.83 | -10.82 | -111,66 |
| 23a | 2.17 ± 0.18 | 5.66 | -11.95 | -106,74 |
| 21a | 2.47 ± 0.22 | 5.61 | -14.09 | -113,74 |
| 19i | 2.7 ± 0.2 | 5.57 | -11.13 | -108,69 |
| 25b | 4.2 ± 0.15 | 5.38 | -12.10 | -105,39 |
| 27c | 14.0 ± 2.0 | 4.85 | -12.00 | -86,74 |
| 19b | 30.3% @ 2 µM | | -12.05 | -108,81 |
| 19c | 20.1% @ 2 µM | | -10.70 | -107,18 |
| 19n | 7% @ 1 µM | | -11.67 | -105,91 |
| 19o | 15% @ 1 µM | | -11.86 | -109,68 |
| 25a | 0% @ 1 µM | | -12.21 | -102,84 |
| 27a | 0% @ 1 µM | | -12.46 | -109,62 |
| 27b | 0% @ 1 µM | | -11.78 | -107,88 |

**Table S5**. The docking scores, binding free energy results of the best model and in vitro data for HDAC3, MODEL7. The compounds were colored based on the cut off values (2 µM $IC_{50}$). Green color < 2 µM, red color > 2 µM. Outlier is colored in cyan.

| HDAC3 | | | | |
|---|---|---|---|---|
| **code** | **HDAC3 $IC_{50}$ (µM) or %inhibition** | **HDAC3-$pIC_{50}$** | **Docking_score** | **GB2_01_Emin1** |

| | | | | |
|---|---|---|---|---|
| 19f | 0.31 ± 0.01 | 6.51 | -10.89 | -59.54 |
| 19m | 0.40 ± 0.02 | 6.40 | -10.27 | -61.74 |
| 23a | 0.40 ± 0.01 | 6.40 | -11.00 | -55.46 |
| 19g | 0.49 ± 0.06 | 6.31 | -10.93 | -64.91 |
| 23b | 0.50 ± 0.02 | 6.30 | -9.63 | -56.37 |
| 19h | 0.57 ± 0.02 | 6.24 | -9.69 | -59.32 |
| 19k | 0.59 ± 0.03 | 6.23 | -10.91 | -60.11 |
| 23c | 0.59 ± 0.04 | 6.23 | -10.26 | -61.13 |
| 19l | 0.81 ± 0.05 | 6.09 | -9.13 | -52.94 |
| 19e | 0.84 ± 0.03 | 6.08 | -10.87 | -60.91 |
| 19d | 1.06 ± 0.04 | 5.97 | -10.63 | -61.55 |
| 19a | 1.12 ± 0.07 | 5.95 | -11.28 | -63.69 |
| 25b | 1.6 ± 0.1 | 5.80 | -10.52 | -53.65 |
| 19j | 1.75 ± 0.06 | 5.76 | -10.47 | -59.57 |
| 19i | 1.9 ± 0.1 | 5.72 | -10.93 | -53.66 |
| 29a | 4.4 ± 0.1 | 5.36 | -5.15 | -21.00 |
| 29b | 6.1 ± 0.7 | 5.21 | -5.69 | -18.72 |
| 29c | 6.7 ± 0.5 | 5.17 | -6.11 | -19.75 |
| 25a | 8.7 ± 0.4 | 5.06 | -11.53 | -43.41 |
| 29d | 12.0 ± 1.0 | 4.92 | -5.19 | -18.48 |
| 27c | 14.0 ± 1.0 | 4.85 | -9.99 | -37.10 |
| 21c | 15 ± 1 | 4.82 | -7.79 | -27.02 |
| 19b | 65.2% @ 2 μM | | -11.47 | -66.39 |
| 19c | 26.8% @ 2 μM | | -10.26 | -60.36 |
| 19n | 13% @ 1 μM | | -10.89 | -55.46 |
| 19o | 30% @ 1 μM | | -11.40 | -55.28 |
| 21a | 0% @ 1 μM | | -6.46 | -15.32 |
| 21b | 0% @ 1 μM | | -7.83 | -29.06 |
| 27a | 0% @ 1 μM | | -8.51 | -44.71 |
| 27b | 0% @ 1 μM | | -11.91 | -43.26 |

**Table S6**. The docking scores, binding free energy results, and prediction results of the test set for HDAC1.

| HDAC1 | | | | | |
|---|---|---|---|---|---|
| **code** | **HDAC1 $IC_{50}$ (μM) or %inhibition** | **HDAC1-$pIC_{50}$** | **Predicted HDAC1 $pIC_{50}$** | **Docking_score** | **GB1_MD-1-50** |
| 30a | 0.32 ± 0.062 | 6.49 | 6.55 | -12.11 | -76.74 |
| 30b | 0.04 ± 0.006 | 7.39 | 6.31 | -12.36 | -73.71 |

| | | | | | |
|---|---|---|---|---|---|
| 30c | 0.019 ± 0.001 | 7.72 | 6.50 | -12.53 | -75.83 |
| 30d | 1.9 ± 0.1 | 5.72 | 5.70 | -9.30 | -60.78 |
| 31a | 4% @1 μM | | 5.58 | -10.85 | -58.50 |
| 31b | 0% @1 μM | | 5.77 | -10.99 | -62.06 |
| 31c | 6% @1 μM | | 5.68 | -10.96 | -60.33 |

**Table S7**. The docking scores, binding free energy results, and prediction results of the test set for HDAC2.

| HDAC2 | | | | | |
|---|---|---|---|---|---|
| **code** | **HDAC2 $IC_{50}$ (μM) or %inhibition** | **HDAC2-$pIC_{50}$** | **Predicted HDAC2 $pIC_{50}$** | **Docking_score** | **GB8_MD-1-50** |
| 30a | 0.61 ± 0.02 | 6.21 | 6.00 | -14.01 | -112.67 |
| 30b | 0.79 ± 0.02 | 6.10 | 6.24 | -14.15 | -118.28 |
| 30c | 1.1 ± 0.1 | 5.96 | 5.95 | -13.95 | -111.56 |
| 30d | 24 ± 2 | 4.62 | 5.89 | -12.01 | -110.06 |
| 31a | 10% @1 μM | | 5.52 | -11.81 | -101.63 |
| 31b | 13% @1 μM | | 5.29 | -11.80 | -96.34 |
| 31c | 14% @1 μM | | 5.52 | -11.78 | -101,56 |

**Table S8**. The docking scores, binding free energy results, and prediction results of the test set for HDAC3.

| HDAC3 | | | | | |
|---|---|---|---|---|---|
| **code** | **HDAC3 $IC_{50}$ (μM) or %inhibition** | **HDAC3-$pIC_{50}$** | **Predicted HDAC3 $pIC_{50}$** | **Docking_score** | **GB2_01_Emin1** |
| 30a | 8% @1 μM | | 5.06 | -5.51 | -23.42 |
| 30b | 6% @1 μM | | 5.01 | -5.23 | -21.48 |
| 30c | 4% @1 μM | | 4.98 | -7.71 | -20.77 |
| 30d | 15% @1 μM | | 5.98 | -10.97 | -54.11 |
| 31a | 21% @1 μM | | 5.81 | -9.76 | -48.42 |
| 31b | 19% @1 μM | | 5.80 | -11.12 | -48.17 |
| 31c | 25% @1 μM | | 5.79 | -11.13 | -47.74 |

## 1. Synthetic Procedures for test set:

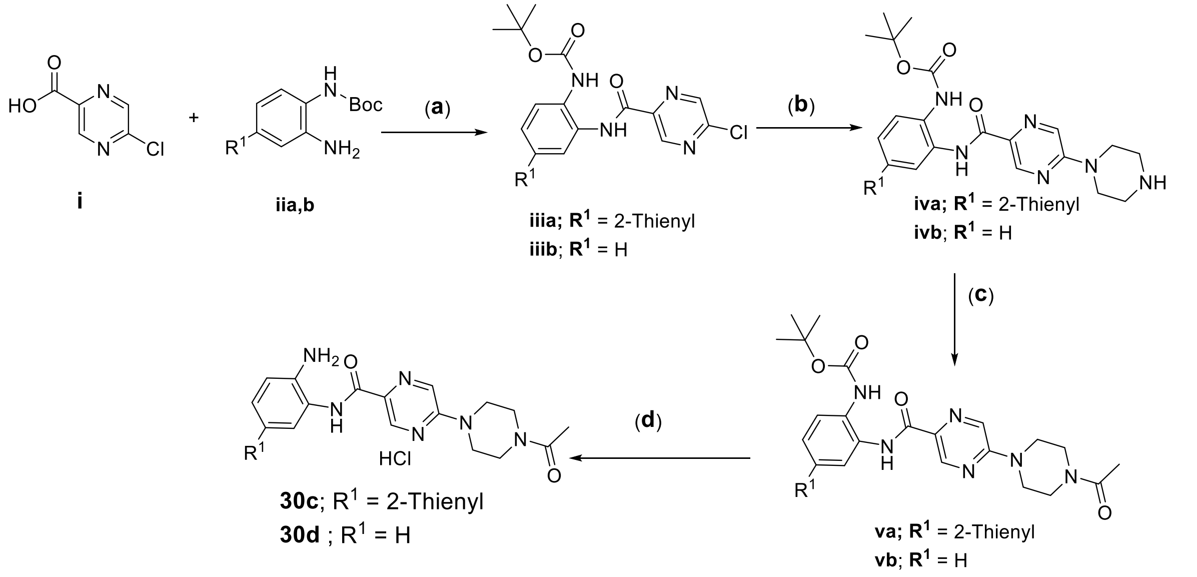


**Scheme S1**. Synthesis of novel compounds **30c** and **30d.** Reagents and conditions: (**a**) HATU, DIPEA, DMF, RT. (**b**) piperazine, toluene, 130 $^{o}$C, 1 h. (**c**) AcCl, TEA, DCM, 0 $^{o}$C, 2 h. (**d)** 4M HCl, dioxane, 0 $^{o}$C, 1 h.

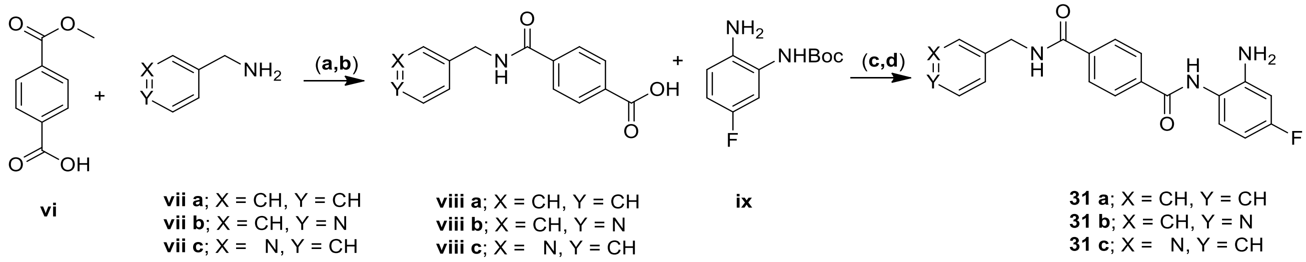


**Scheme S2**. Synthesis of compounds **31a-c.** Reagents and conditions: (**a**) EDCI, DMF, 18 h, RT. (**b**) LiOH.$H_2O$, THF, $H_2O$, 2 h (**c**) HATU, DIPEA, DMF, 18 h, RT. (**d)** TFA, DCM, 0 $^{o}$C, 1 h.

### *1.1.* General specifications for materials and methods:

Materials and reagents were purchased from Sigma-Aldrich Co. Ltd. and abcr GmbH. All solvents were analytically pure and dried before use. Thin layer chromatography was carried out on aluminum sheets coated with silica gel 60 F254 (Merck, Darmstadt, Germany). For medium pressure chromatography (MPLC) silica gel 60 (0.036e0.200 mm) was used. Final compounds were confirmed to be of >95% purity based on HPLC.

Purity was measured by UV absorbance at 254 nm. The HPLC consists of an XTerra RP18 column (3.5 µm, 3.9 mm x 100 mm) from the manufacturer Waters (Milford, MA, USA) and two LC-10AD pumps, a SPD-M10A VP PDA detector, and a SIL-HT autosampler, all from the manufacturer Shimadzu (Kyoto, Japan). Mass spectrometry analyses were performed with a Finnigan MAT710C (Thermo Separation Products, San Jose, CA, USA) for the ESIMS spectra and with a LTQ (linear ion trap) Orbitrap XL hybrid mass spectrometer (Thermo Fisher Scientific, Bremen, Germany). $^{1}$H NMR spectra were taken on a Varian Inova 400 using deuterated DMSO as solvent. Chemical shifts are referenced to the residual solvent signals.

### *1.2.* General method for synthesis of intermediates (iiia) and (iiib):

5-chloropyrazine-2-carboxylic acid (**i**; 1mmol; 0.15 g) was mixed with O-(7-Azabenzotriazol-1-yl)-*N,N,N',N'*-tetramethyluronium-hexafluorphosphat (HATU) (1.2 mmol; 0.46 g) and the appropriate amines (**iia,b**; 1 mmol) in 10 ml DMF followed by the addition of of DIPEA (4 mmol; 0.517 g) at room temperature. The reaction was followed by TLC till the full consumption of the starting materials (18 h for **iiia** and 2 h for **iiib**). The reaction mixture was poured on ice and extracted with ethyl acetate. The separated organic layer was washed by 2M $NH_4Cl$ and then 1M $Na_2CO_3$. The organic layer was dried, concentrated, and then the residue was purified by recrystallization from ethyl acetate.

*Tert-butyl (2-(5-chloropyrazine-2-carboxamido)-4-(thiophen-2-yl)phenyl)carbamate **(iiia)***

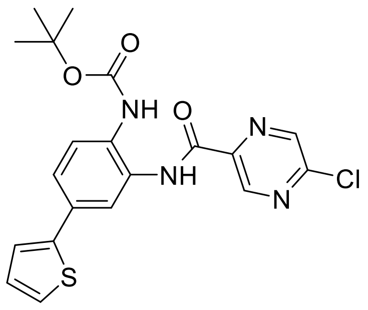


$^{1}$H NMR (400 MHz, $CDCl_3$-*d*) δ 9.99 (s, 1H), 8.89 (s, 1H), 8.73 (d, J = 4.6 Hz, 2H), 8.51 (d, J = 9.7 Hz, 1H), 8.07 (s, 1H), 7.50 (dd, J = 8.4, 4.5 Hz, 1H), 7.45 (d, J = 1.9 Hz, 1H),

7.30 (dd, J = 3.6, 0.7 Hz, 1H), 7.06 (dd, J = 5.0, 3.7 Hz, 1H), 1.56 (s, 9H)., MS m/z: 331,4 (19%), $[M+H]^+$, Yield: 68 %.

*Tert-butyl (2-(5-chloropyrazine-2-carboxamido)phenyl)carbamate (**iiib**)*

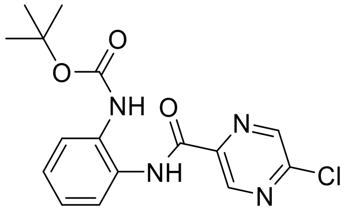

$^1$H NMR (400 MHz, DMSO-$d_6$) δ 10.29 (s, 1H), 9.14 (d, J = 1.3 Hz, 1H), 8.87 – 8.70 (m, 2H), 7.82 (dd, J = 7.6, 1.9 Hz, 1H), 7.67 (dd, J = 8.4, 4.5 Hz, 1H), 7.33 (dd, J = 7.5, 2.0 Hz, 1H), 7.19 (d, J = 2.3 Hz, 1H), 1.50 (s, 9H), MS m/z: 349.3 (83%) $[M+H]^+$, Yield: 64 %.

### *1.3.* General method for synthesis of intermediates (iva) and (ivb):

Compound **iiia** or **iiib** (1 mmol) was dissolved with piperazine (0.003mol; 0.26 g) in 30 ml toluene and the heated at 130 $^0$C for 1h. The reaction mixture was poured into ice and then extracted with $CHCl_3$. The organic layer was separated, dried and concentrated. The obtained solid was washed different times with hexane to get rid of residual toluene. The resulting product was proceeded to the next step directly without any further purification.

*Tert-butyl(2-(5-(piperazin-1-yl)pyrazine-2-carboxamido)-4-(thiophen-2-yl)phenyl)carbamate (**iva**)*

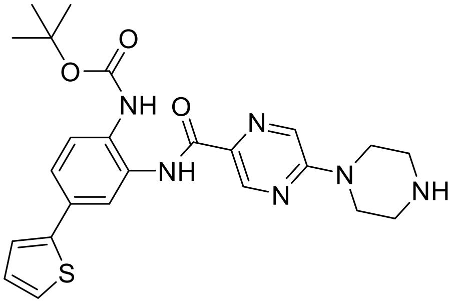

$^{1}H$ NMR (400 MHz, DMSO-$d_6$) δ 10.00 (s, 1H), 9.05 (s, 1H), 8.72 (d, $J$ = 1.2 Hz, 1H), 8.21 (s, 1H), 7.53 (dd, $J$ = 5.1, 1.0 Hz, 1H), 7.46 – 7.40 (m, 2H), 7.29 (d, $J$ = 8.4 Hz, 1H), 7.25 – 7.20 (m, 1H), 7.17 – 7.09 (m, 2H), 3.88 – 3.48 (m, 4H), 2.86 – 2.69 (m, 4H), 1.47 (s, 9H), Yield: 50 %.

*Tert-butyl (2-(5-(piperazin-1-yl)pyrazine-2-carboxamido)phenyl)carbamate (**ivb**)*

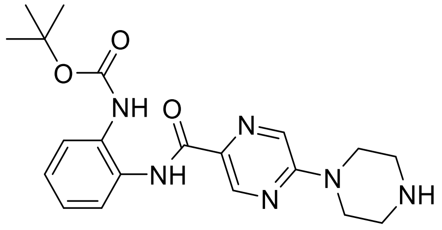


$^{1}H$ NMR (400 MHz, DMSO-$d_6$) δ 10.01 (s, 1H), 9.33 (s, 1H), 9.02 (s, 1H), 8.77 (d, J = 1.3 Hz, 1H), 8.32 (s, 1H), 7.94 (d, J = 7.3 Hz, 1H), 7.22 (m, 2H), 7.12 (td, J = 7.6, 1.5 Hz, 1H), 4.10 – 3.80 (m, 4H), 3.23 – 3.11 (m, 4H), 1.47 (s, 9H), MS m/z: 399.3 (100%) $[M+H]^+$, 397.4 (66%) $[M-H]^+$, Yield: 54 %.

## *1.4.* General method for synthesis of intermediates (va) and (vb)

Compound **va** or **vb** (1 mmol) was dissolved with TEA (3 mmol; 0.30 g) in 30 ml DCM and stirred for 30 min at 0 $^{o}$C. Acetyl chloride (1.5 mmol; 0.12 g) was added dropwise to the previous mixture then the reaction was followed by TLC till the full consumption of the starting materials. The reaction mixture was washed with 10% citric acid and then the organic layer was separated, dried and concentrated. The obtained solid was subjected to purification by MPLC (DCM:Meth).

*Tert-Butyl(2-(5-(4-acetylpiperazin-1-yl)pyrazine-2-carboxamido)-4-(thiophen-2-yl)phenyl)carbamate (**va**)*

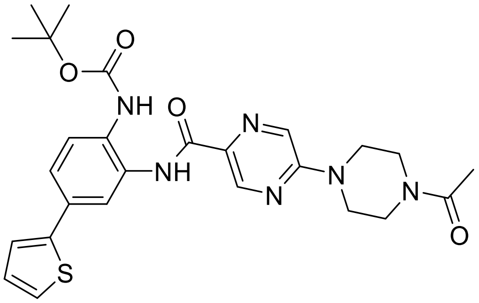

$^{1}H$ NMR (400 MHz, $CDCl_3$-*d*) δ 9.63 (s, 1H), 8.97 (d, J = 1.2 Hz, 1H), 8.01 (d, J = 1.1 Hz, 1H), 7.92 (s, 1H), 7.61 (d, J = 8.0 Hz, 1H), 7.45 (dd, J = 8.4, 2.1 Hz, 1H), 7.33 – 7.29 (m, 1H), 7.27 (d, J = 1.0 Hz, 1H), 7.17 (dd, J = 12.3, 4.8 Hz, 1H), 7.06 (dd, J = 5.1, 3.6 Hz, 1H), 3.86 – 3.77 (m, 4H), 3.74 – 3.69 (m, 2H), 3.64 – 3.60 (m, 2H), 2.16 (s, 3H), 1.51 (s, 9H), MS m/z: 423.3 (57%), 523.4 (78%), $[M+H]^+$, Yield: 48 %.

*tert-Butyl (2-(5-(4-acetylpiperazin-1-yl)pyrazine-2-carboxamido)phenyl)carbamate (**vb**)*

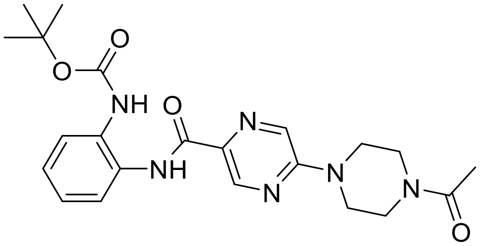

$^{1}H$ NMR (400 MHz, $CDCl_3$-*d*) δ 9.61 (s, 1H), 8.96 (d, J = 1.3 Hz, 1H), 8.00 (d, J = 1.3 Hz, 1H), 7.66 (d, J = 5.3 Hz, 1H), 7.57 (d, J = 7.1 Hz, 1H), 7.24 – 7.16 (m, 2H), 7.11 (s, 1H), 3.85 – 3.77 (m, 4H), 3.71 (m, 2H), 3.65 – 3.61 (m, 2H), 2.16 (s, 23H), 1.51 (s, 9H)., MS m/z: 341,3 (100%), 441.4 (92%) $[M+H]^+$, 440.4 (20%) $[M-H]^+$, Yield: 51 %.

### *1.5.* **General method for synthesis of intermediates viii a-c**

A mixture of 4-(methoxycarbonyl)benzoic acid (**vi**; 1.0 eq.), the corresponding amine (**vii a-c; 1 eq.**) and EDCI (1.5 eq.) and were dissolved in dry DMF (5 mL) and stirred at room temperature for 18 h. EtOAc (20 mL) was added and the mixture was washed with 2N $NH_4Cl$. The organic extract was washed with brine, dried over anhydrous $Na_2SO_4$, filtered and the solvent was removed under reduced pressure. The residue was purified by using MPLC ($CHCl_3$: MeOH) to provide the corresponding ester. This ester was treated with $LiOH.H_2O$ (4 eq.) in $H_2O$ (5 mL) and THF (5 mL) and left stirring at room

temperature for 2 h. Then HCl (1N) was added till pH 5. The formed solid was filtered and dried to obtain the corresponding carboxylic acid (**viii a-c**). Reaction yields, spectral data are reported below.

*4-(Benzylcarbamoyl)benzoic acid* (**viiia**)

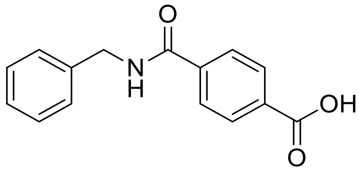

$^{1}$H NMR (400 MHz, DMSO-$d_6$) δ 13.02 (s, 1H), 9.17 (t, *J* = 6.0 Hz, 1H), 8.02 – 7.88 (m, 5H), 7.38 - 7.31 (m, 4H), 4.48 (d, *J* = 6.0 Hz, 2H). Yield: 35% over 2 steps.

*4-((Pyridin-4-ylmethyl)carbamoyl)benzoic acid* ***(viiib)***

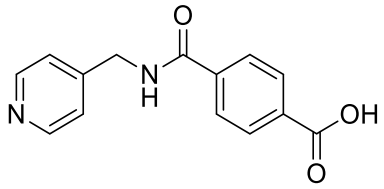

$^{1}$H NMR (400 MHz, DMSO-$d_6$) δ 13.22 (s, 1H), 9.30 (t, *J* = 5.9 Hz, 1H), 8.51 (dd, *J* = 4.5, 1.6 Hz, 2H), 8.06 – 7.95 (m, 4H), 7.34 (d, *J* = 6.0 Hz, 2H), 4.51 (d, *J* = 5.9 Hz, 2H). 61% over 2 steps.

*4-((Pyridin-3-ylmethyl)carbamoyl)benzoic acid* ***(viiic)***

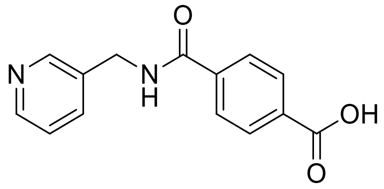

$^{1}H$ NMR (400 MHz, DMSO-$d_6$) δ 13.18 (s, 1H), 9.23 (t, *J* = 5.9 Hz, 1H), 8.55 (s, 1H), 8.44 – 8.38 (m, 1H), 7.98 (dd, *J* = 19.9, 8.5 Hz, 5H), 7.35 (dd, *J* = 7.2, 4.7 Hz, 1H), 4.50 (d, *J* = 5.6 Hz, 2H). Yield: 61% over 2 steps.

## *1.6.* **General method for synthesis of final compounds**

### *1.6.1.* ***General method for synthesis of final compounds*** **(30a,b)**

Compounds (**30a,b)** were prepared using the same procedures as reported **[1].**

*N-(2-Amino-5-(thiophen-2-yl)phenyl)-2-(4-methylpiperazin-1-yl)pyrimidine-5-carboxamide* **(30a)**

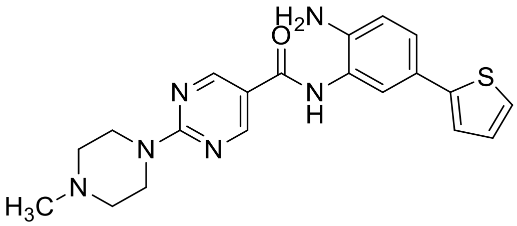

$^{1}H$ NMR (400 MHz, DMSO-$d_6$) δ 9.53 (s, 1H), 8.89 (s, 2H), 7.44 (d, *J* = 2.1 Hz, 1H), 7.33 (dd, *J* = 5.1, 1.1 Hz, 1H), 7.27 (dd, *J* = 8.3, 2.2 Hz, 1H), 7.22 (dd, *J* = 3.6, 1.1 Hz, 1H), 7.03 (dd, *J* = 5.1, 3.6 Hz, 1H), 6.78 (d, *J* = 8.4 Hz, 1H), 5.18 (s, 2H), 3.89 – 3.79 (m, 4H), 2.40 – 2.33 (m, 4H), 2.21 (s, 3H). $^{13}C$ NMR (101 MHz, dmso) δ 163.26, 161.87, 158.61, 144.68, 143.60, 128.64, 124.51, 124.43, 123.60, 123.30, 122.50, 121.39, 116.64, 116.54, 54.79, 46.19, 43.89. MS m/z: 395.25 $[M+H]^+$, HPLC: rt 7.44 min (purity 99.82%), Yield: 88%.

*N-(2-Amino-5-(thiophen-2-yl)phenyl)-5-(4-methylpiperazin-1-yl)pyrazine-2-carboxamide* **(30b)**

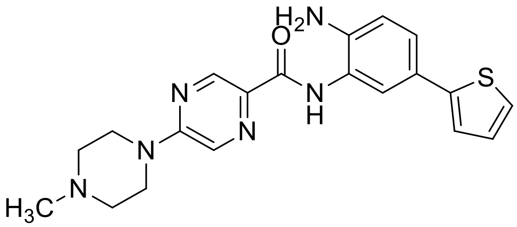

$^{1}H$ NMR (400 MHz, DMSO-$d_6$) δ 9.66 (s, 1H), 8.71 (d, *J* = 1.3 Hz, 1H), 8.35 (d, *J* = 1.2 Hz, 1H), 7.77 (d, *J* = 2.1 Hz, 1H), 7.35 (dd, *J* = 5.1, 1.0 Hz, 1H), 7.24 (ddd, *J* = 6.0, 4.7, 1.6 Hz, 2H), 7.04 (dd, *J* = 5.1, 3.6 Hz, 1H), 6.83 (d, *J* = 8.3 Hz, 1H), 5.07 (s, 2H), 3.78 – 3.65 (m, 4H), 2.46 – 2.35 (m, 4H), 2.22 (s, 3H). MS m/z: 395.38 $[M+H]^+$, HPLC: rt 7.71 min (purity 100.0%), Yield: 85%.

### *1.6.2.* **General method for synthesis of final compounds (30c) and (30d)**

0.1 g of the *N*-BOC protected intermediates (**va**) and (**vb**) were dissolved in 10 ml dioxane and stirred at 0 $^{0}$C, and then 5 ml of 4M HCl in dioxane was added dropwise. The reaction mixture was stirred for 2 h till appearance of a heavy precipitate. The precipitate was filtered, dried and washed many times with hexane to get rid of residual HCl. The analytical data of the products are given below.

*5-(4-acetylpiperazin-1-yl)-N-(2-amino-5-(thiophen-2-yl)phenyl)pyrazine-2-carboxamide.HCl (**30c**)*

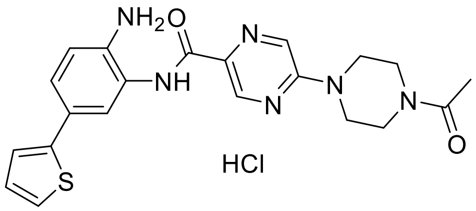

$^{1}H$ NMR (400 MHz, DMSO-$d_6$) δ 10.41 (s, 1H), 8.76 (d, J = 1.2 Hz, 1H), 8.38 (d, J = 1.2 Hz, 1H), 7.97 – 7.86 (m, 3H), 7.63 – 7.56 (m, 3H), 7.52 (dd, J = 3.6, 1.0 Hz, 1H), 7.14 (dd, J = 5.1, 3.6 Hz, 1H), 3.85 – 3.79 (m, 2H), 3.77 – 3.72 (m, 2H), 3.61 – 3.55 (m, 4H), 2.05 (s, 3H), $^{13}C$ NMR (101 MHz, DMSO-$d_6$) δ 169.04, 163.52, 162.74, 155.44, 142.98, 142.23, 133.64, 132.52, 132.27, 129.40, 129.09, 126.90, 124.95, 123.97, 123.54, 45.38,

44.30, 21.71. MS m/z: 341.3 (100%), $[M+H]^+$, HPLC: rt 11.964 min (purity 97.8 %) Yield: 72 %.

*5-(4-Acetylpiperazin-1-yl)-N-(2-aminophenyl)pyrazine-2-carboxamide.HCl (**30d**)*

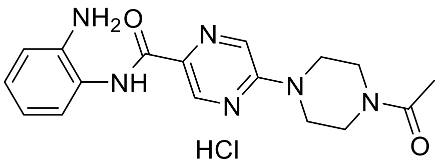

$^1$H NMR (400 MHz, DMSO-$d_6$) δ 10.29 (s, 1H), 8.74 (d, J = 1.0 Hz, 1H), 8.36 (d, J = 1.0 Hz, 1H), 7.60 (dd, J = 7.8, 1.2 Hz, 1H), 7.46 (d, J = 7.6 Hz, 1H), 7.37 – 7.22 (m, 2H), 3.85 – 3.78 (m, 2H), 3.76 – 3.71 (m, 2H), 3.61 – 3.56 (m, 4H), 2.05 (s, 3H), $^{13}$C NMR (101 MHz, DMSO-$d_6$) δ 169.02, 163.33, 155.44, 142.87, 132.69, 131.08, 129.37, 127.24, 126.73, 123.69, 45.38, 44.30, 44.10, 21.72. MS m/z: 341.3 (100%), $[M+H]^+$, HPLC: rt 8.473 min (purity 99.7 %) Yield: 75 %.

### *1.6.3.* **General method for synthesis of final compounds (31a-c)**

A mixture of the appropriate carboxylic acid (**viii a-c**; 1.0 eq.) and HATU (1.2 eq.) was dissolved in dry DMF (5 mL) and stirred at room temperature for 30 min. The corresponding amine (**ix**; 0.9 eq.) and DIPEA (5.0 eq.) in THF (3 mL) were added and the reaction mixture was stirred for 18 h at room temperature. The reaction mixture was diluted with EtOAc (15 mL) and the reaction mixture was washed with 1N $NH_4Cl$ followed by saturated $NaHCO_3$. The organic extracts were washed with brine, dried over anhydrous $Na_2SO4$, filtered and the organic solvents were evaported under vacuum. The residue was purified by using MPLC ($CHCl_3$:MeOH) to provide the corresponding amide. The obtained product was dissolved in dry DCM (5 mL), and then TFA (5 mL) was added. The reaction mixture was stirred at room temperature for 1 h. The solvent was evaporated to dryness, the residue was dissolved in 1 mL MeOH, 1N NaOH (10 mL) was added, and the mixture was stirred for 1 h, before being extracted with EtOAc. The organic extracts were washed with brine, dried over anhydrous $Na_2SO_4$, filtered and concentrated under vacuum. The residue was purified by using MPLC ($CHCl_3$:MeOH) to

provide the corresponding amide. Reaction yields, chromatographic and spectrometric data of the final compounds are reported below.

*N¹-(2-Amino-4-fluorophenyl)-N⁴-benzylterephthalamide* **(31a)**

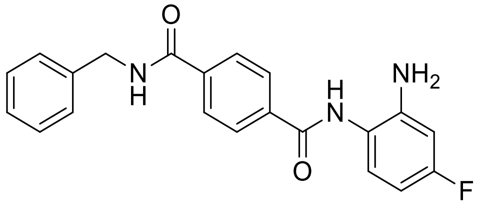

$^{1}$H NMR (400 MHz, DMSO-$d_6$) δ 9.68 (s, 1H), 9.17 (t, *J* = 5.9 Hz, 1H), 8.09 – 7.96 (m, 4H), 7.32 (dd, *J* = 6.8, 3.0 Hz, 4H), 7.29 – 7.19 (m, 1H), 7.12 (dd, *J* = 8.6, 6.4 Hz, 1H), 6.54 (dd, *J* = 11.2, 2.9 Hz, 1H), 6.35 (td, *J* = 8.5, 2.9 Hz, 1H), 5.25 (s, 2H), 4.50 (d, *J* = 6.0 Hz, 2H). $^{13}$C NMR (101 MHz, dmso) δ 165.98, 165.43, 162.75, 160.38, 145.95, 139.94, 137.30, 137.11, 128.75, 128.26, 127.71, 127.58, 127.24, 119.43, 102.55, 102.32, 101.97, 101.72, 43.17. MS m/z: 362.26 [M-H]$^-$. HPLC: rt 11.085 min (purity 97.33%) Yield: 53% over 2 steps.

*N$^1$-(2-Amino-4-fluorophenyl)-N$^4$-(pyridin-4-ylmethyl)terephthalamide* **(31b)**

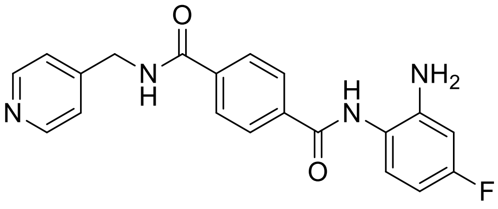

$^{1}$H NMR (400 MHz, DMSO-$d_6$) δ 9.68 (s, 1H), 9.22 (t, *J* = 5.8 Hz, 1H), 8.56 (d, *J* = 1.9 Hz, 1H), 8.46 (dd, *J* = 4.7, 1.4 Hz, 1H), 8.06 (d, *J* = 8.3 Hz, 2H), 7.98 (d, *J* = 8.4 Hz, 2H), 7.73 (d, *J* = 7.8 Hz, 1H), 7.36 (dd, *J* = 7.8, 4.8 Hz, 1H), 7.11 (dd, *J* = 8.6, 6.4 Hz, 1H), 6.53 (dd, *J* = 11.2, 2.9 Hz, 1H), 6.35 (td, *J* = 8.5, 2.8 Hz, 1H), 5.25 (s, 2H), 4.51 (d, *J* = 5.8 Hz, 2H). MS m/z: 365.28 [M+H]$^+$. HPLC: rt 7.66 min (purity 99.72%) Yield: 61% over 2 steps.

*N$^1$-(2-Amino-4-fluorophenyl)-N$^4$-(pyridin-3-ylmethyl)terephthalamide* **(31c)**

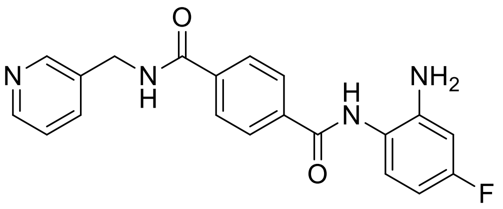

$^{1}$H NMR (400 MHz, DMSO-$d_6$) δ 9.73 (d, *J* = 15.0 Hz, 1H), 9.28 (s, 1H), 8.54 (d, *J* = 14.6 Hz, 2H), 8.06 (d, *J* = 7.6 Hz, 3H), 7.35 (d, *J* = 15.1 Hz, 3H), 7.13 (s, 1H), 6.57 (s, 1H), 6.37 (s, 1H), 5.27 (s, 2H), 4.53 (s, 2H). $^{13}$C NMR (101 MHz, dmso) δ 166.31, 165.41, 160.38, 150.01, 148.84, 146.07, 137.47, 136.78, 129.14, 128.32, 127.62, 122.60, 119.41, 102.55, 102.33, 101.97, 101.72, 42.28. MS m/z: 365.15 $[M+H]^{+}$. HPLC: rt 7.47 min (purity 99.24%) Yield: 61% over 2 steps.

## $^{1}$H NMR and $^{13}$C NMR spectra

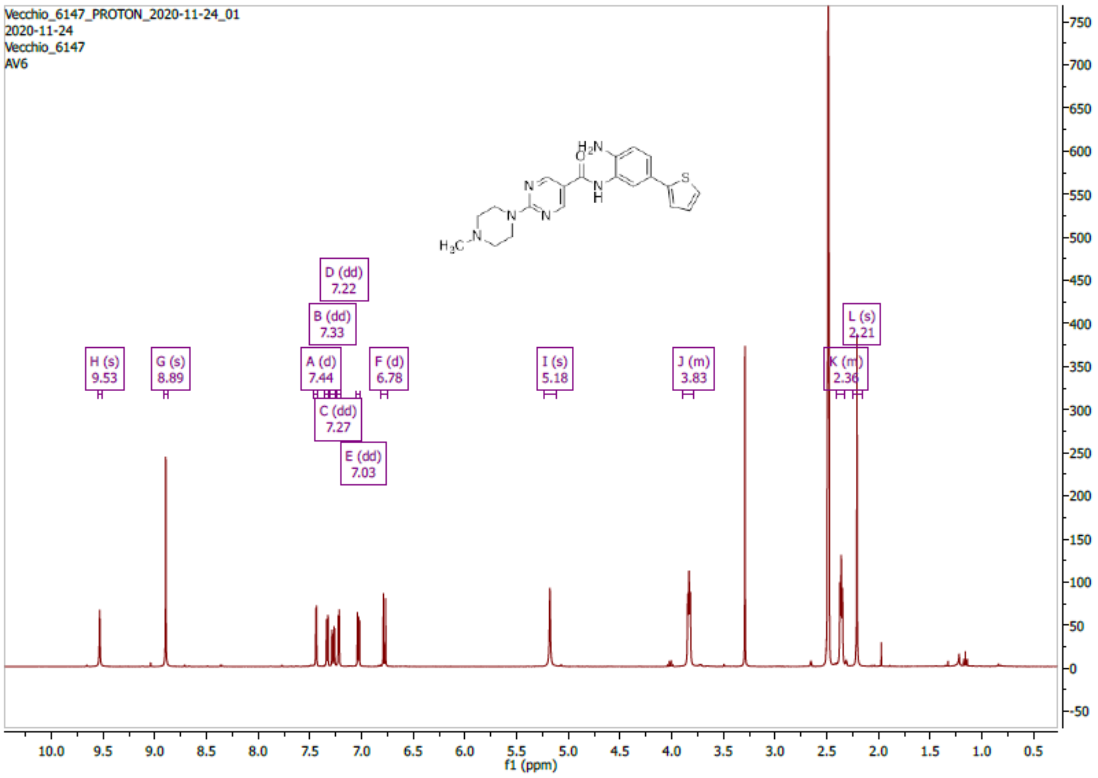

$^{1}$H NMR chart of compound **30a**.

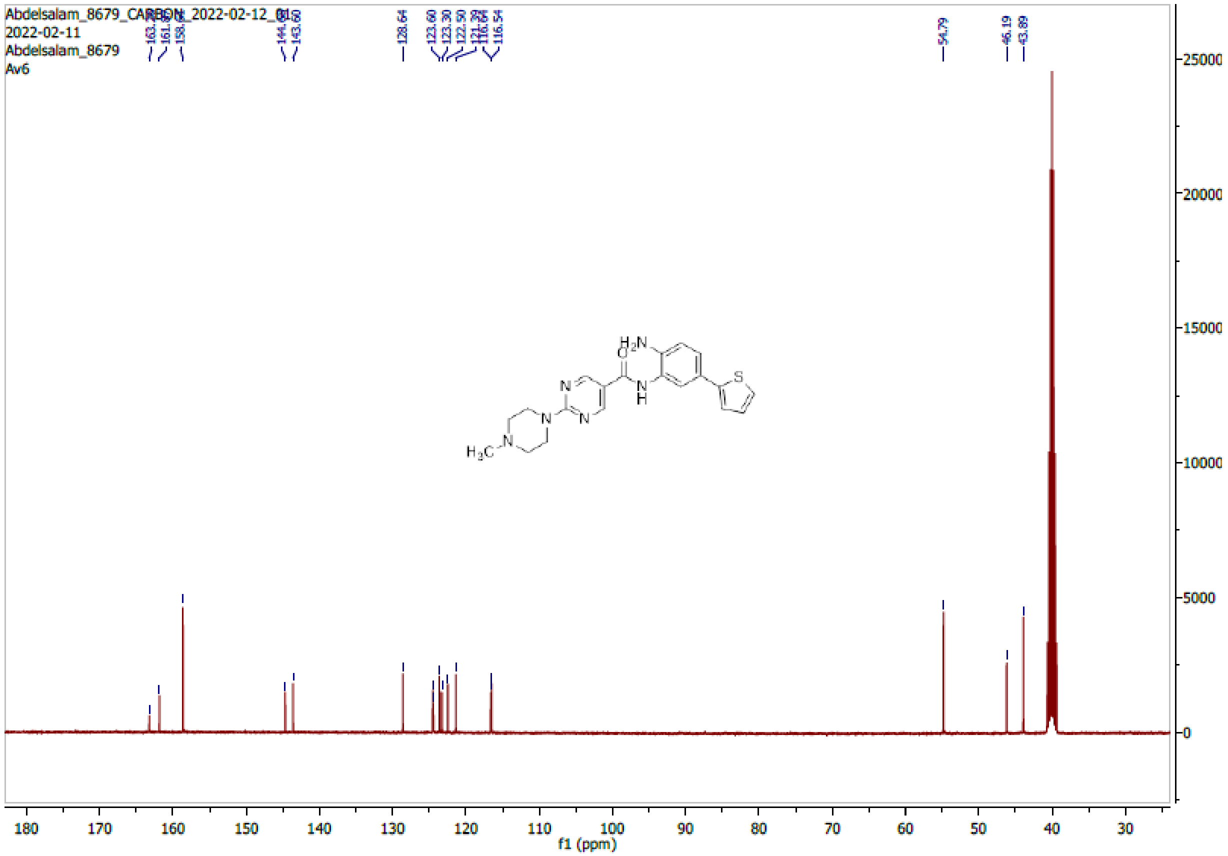

$^{13}$C NMR chart of compound **30a**.

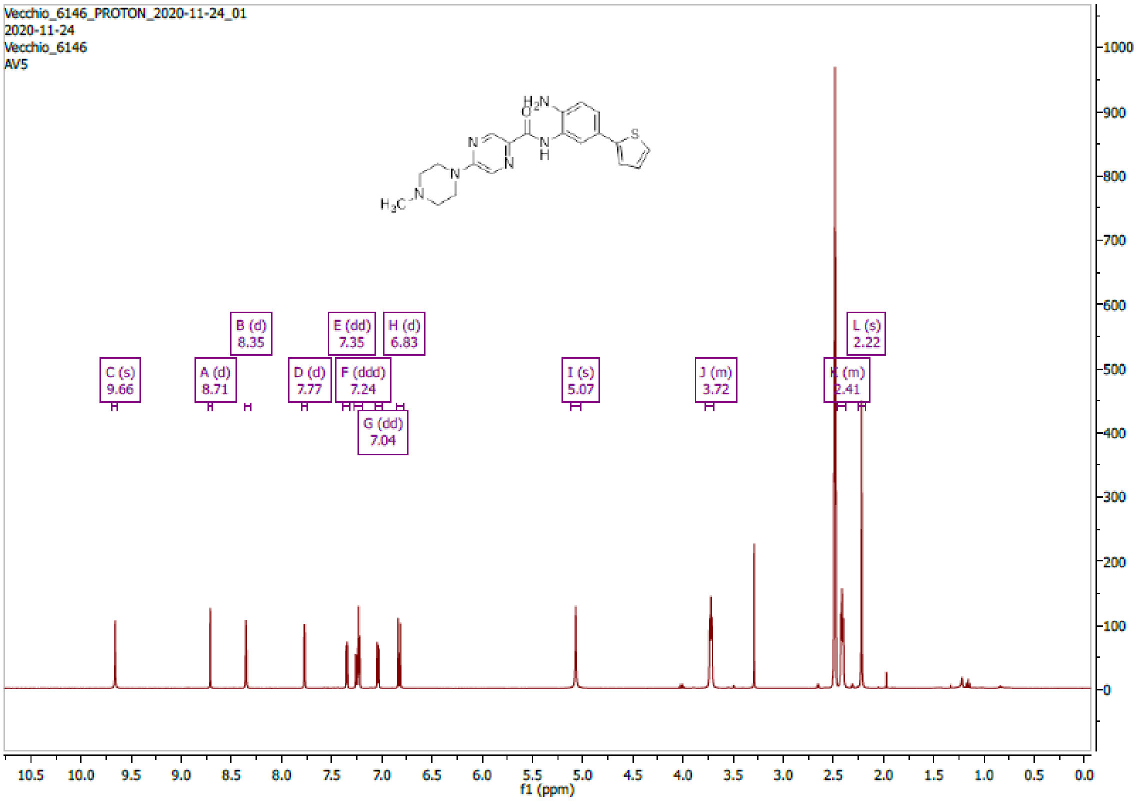


$^{1}$H NMR chart of compound **30b**.

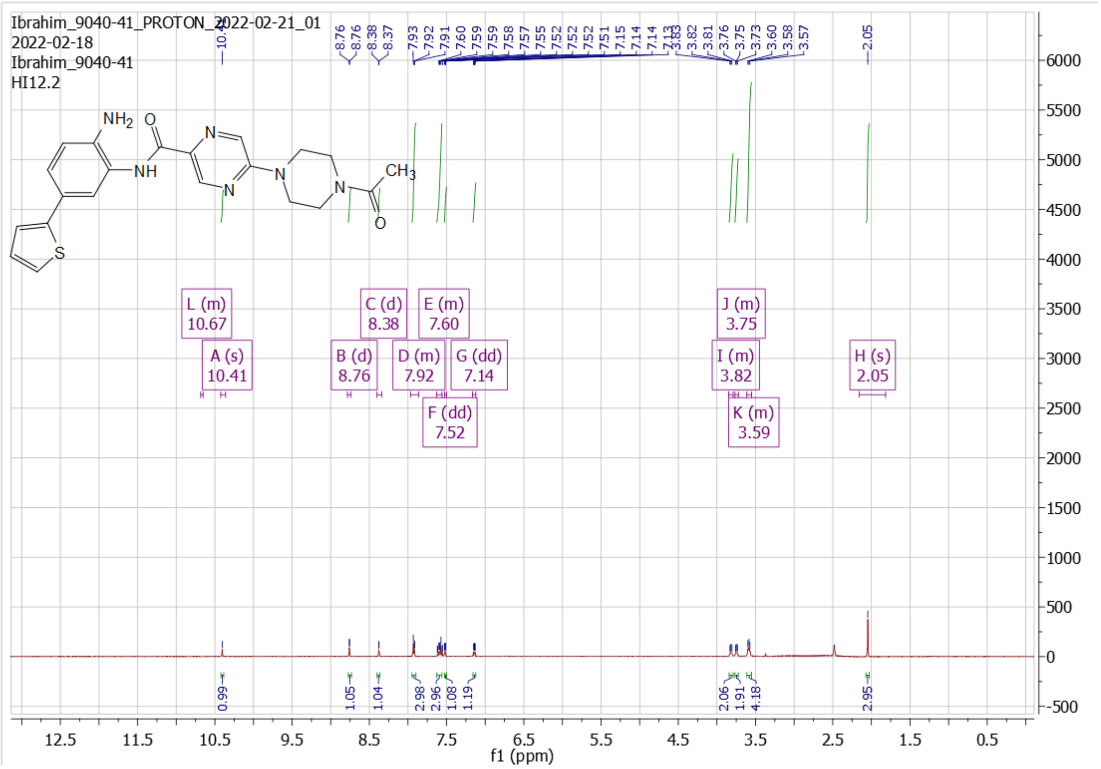

$^{1}$H NMR chart of compound **30c**.

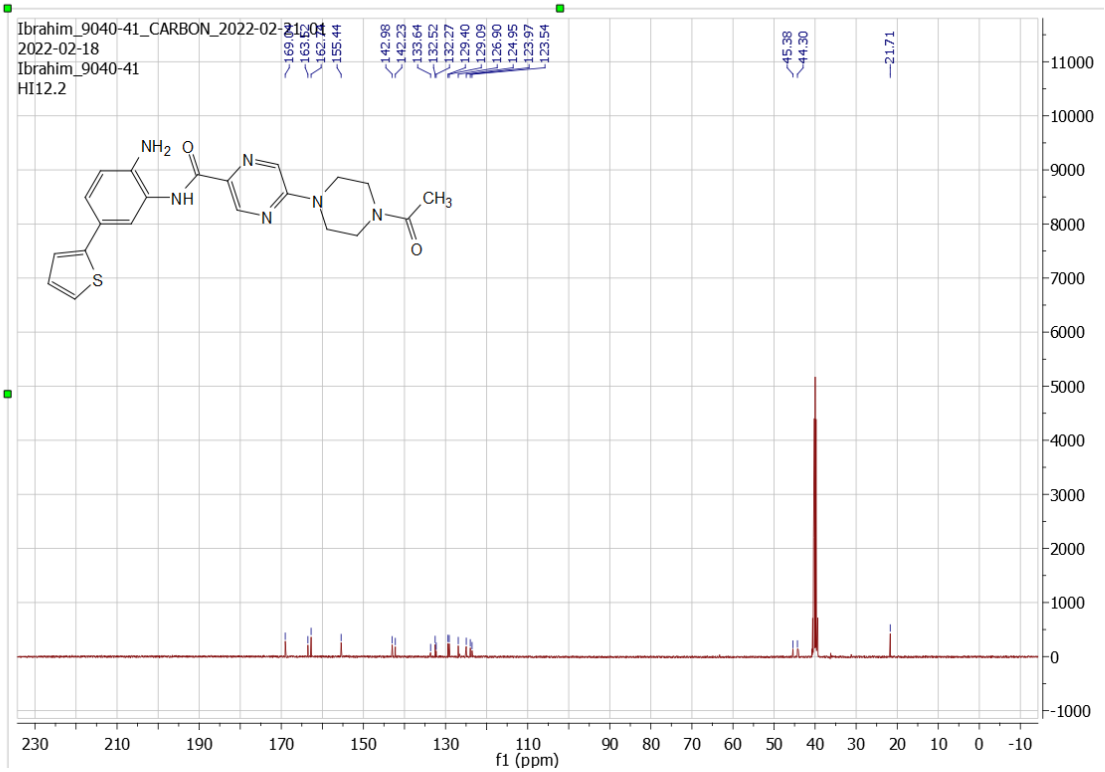


$^{13}$C NMR chart of compound **30c**.

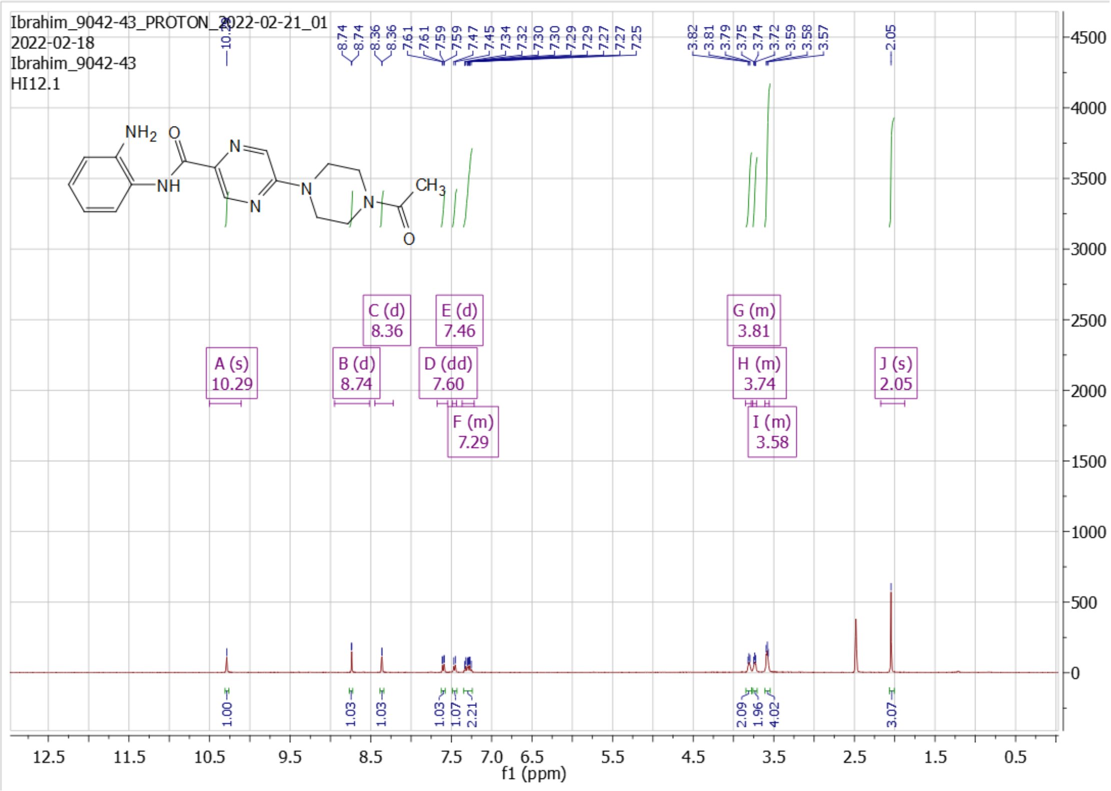

$^{1}$H NMR chart of compound **30d**.

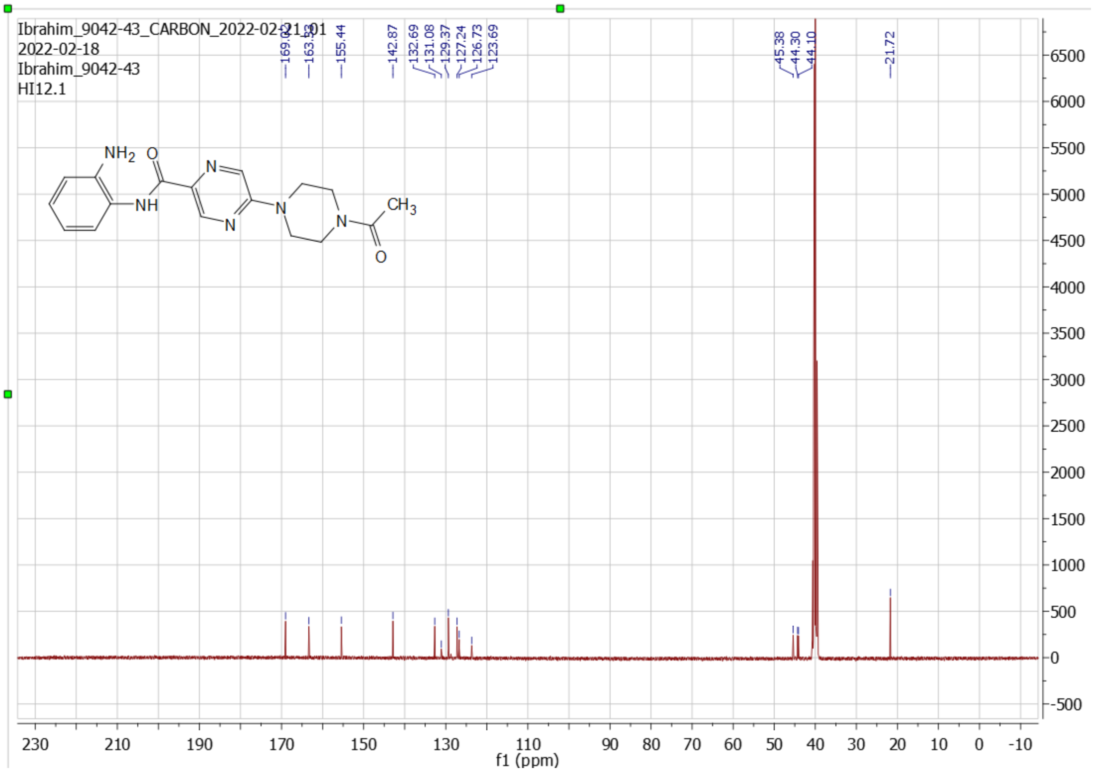

$^{13}$C NMR chart of compound **30d**.

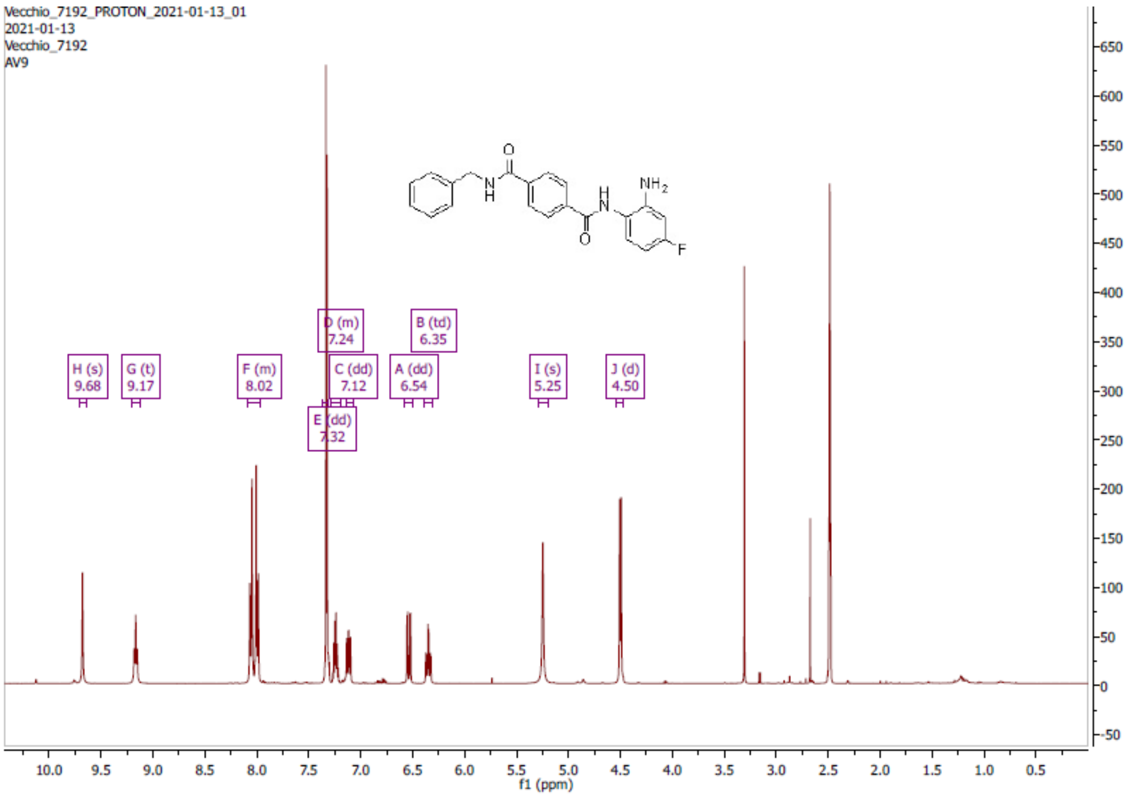

$^{1}$H NMR chart of compound **31a**.

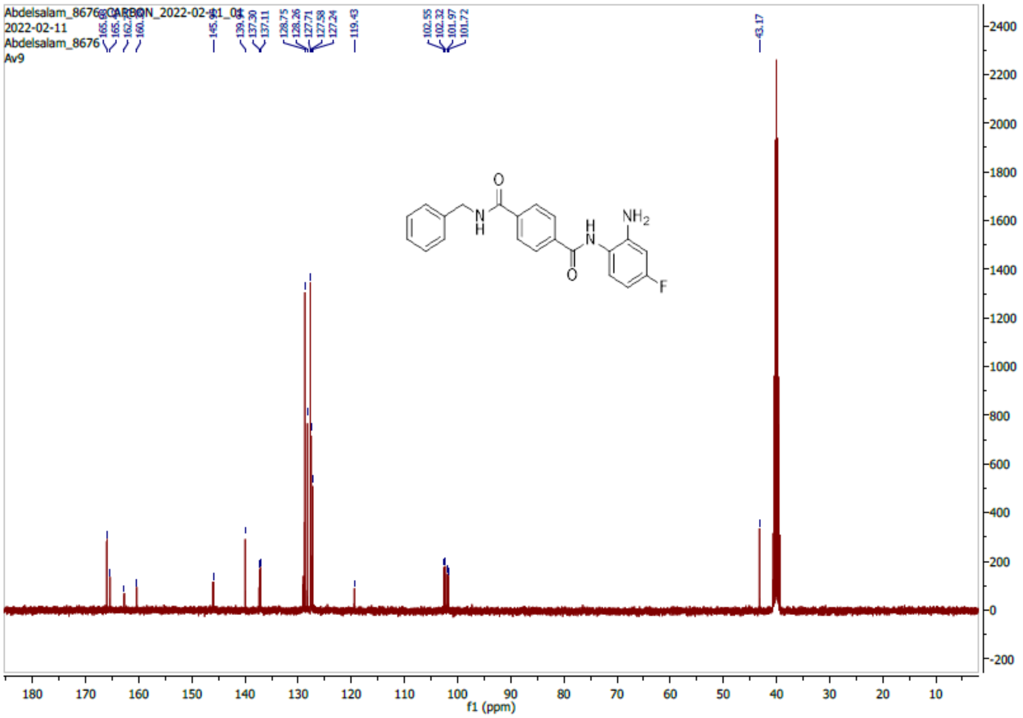

$^{13}$C NMR chart of compound **31a**.

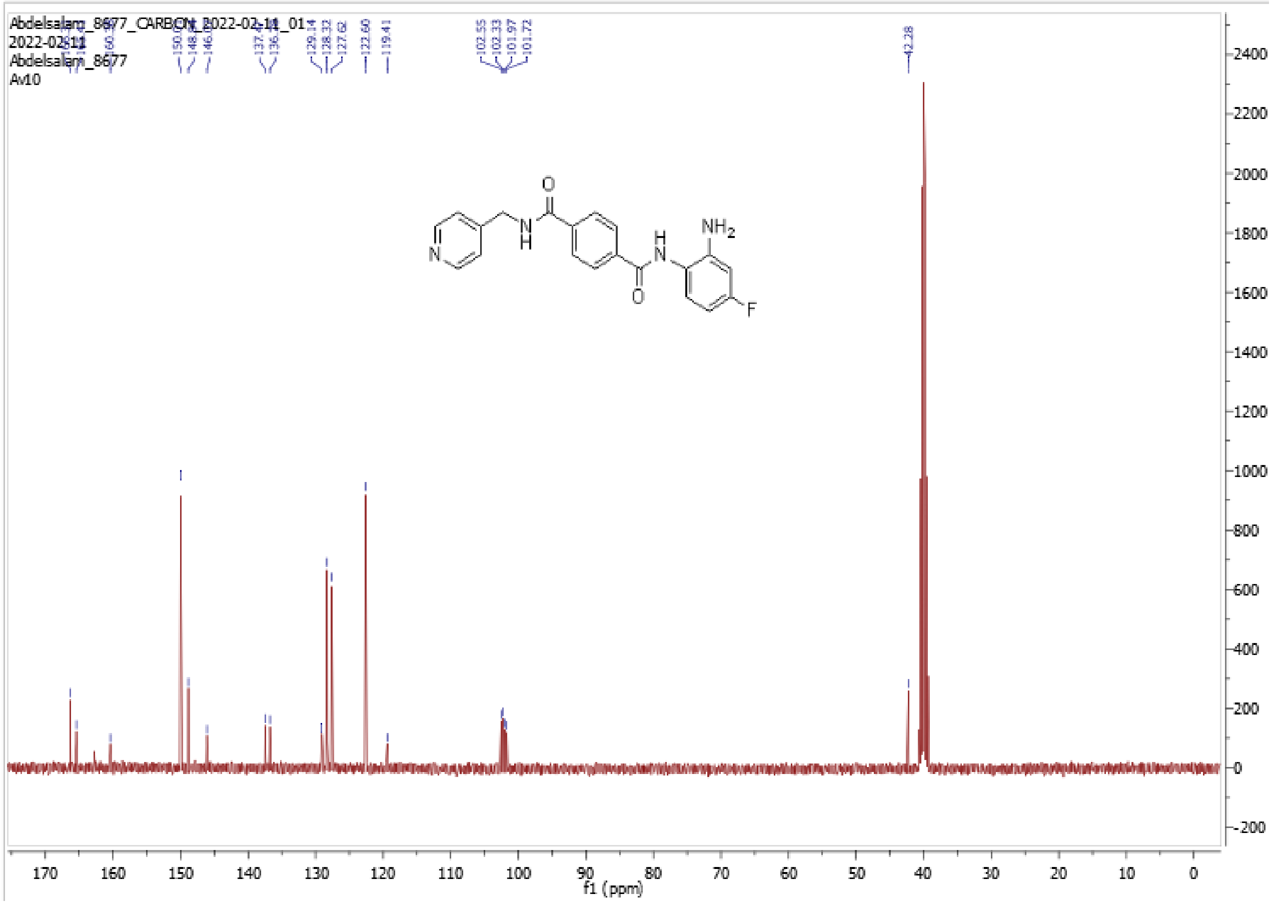

$^{13}$C NMR chart of compound **31b**.

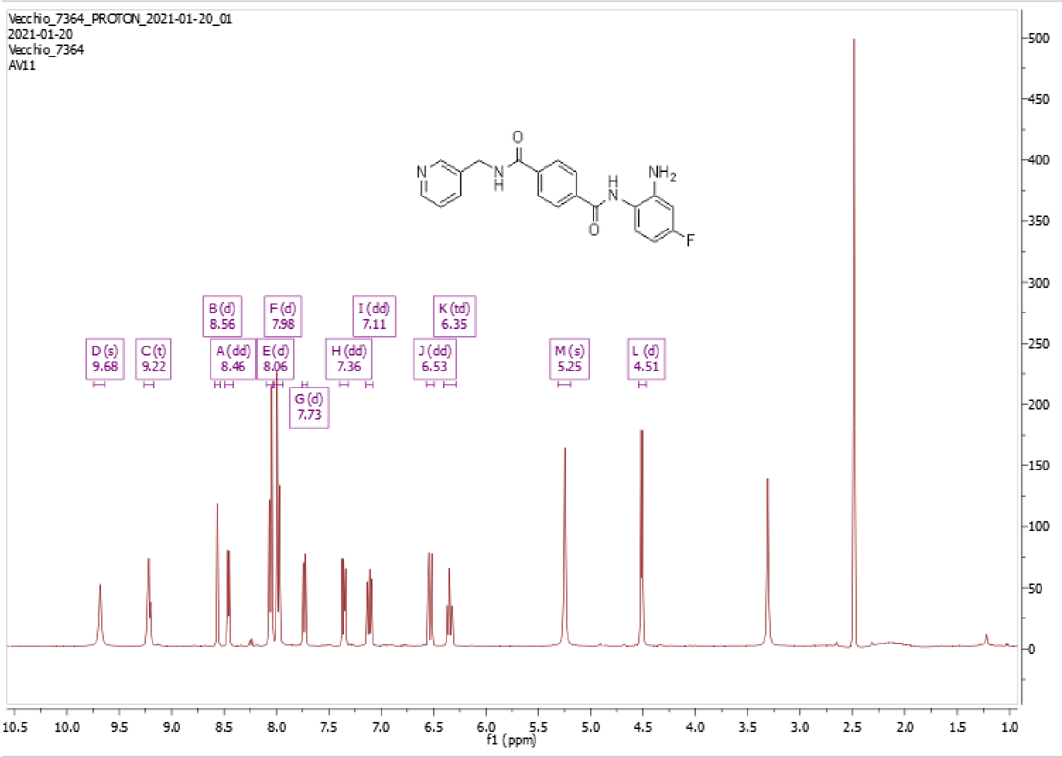


$^1$H NMR chart of compound **31c**.

## References


1. Ibrahim, H.S., et al., *Synthesis, Molecular Docking and Biological Characterization of Pyrazine Linked 2-Aminobenzamides as New Class I Selective Histone Deacetylase (HDAC) Inhibitors with Anti-Leukemic Activity.* Int J Mol Sci, 2021. **23**(1), 369.
